# *In situ* forming and biocompatible hyaluronic acid hydrogel with reactive oxygen species-scavenging activity to improve traumatic brain injury repair by suppressing oxidative stress and neuroinflammation

**DOI:** 10.1016/j.mtbio.2022.100278

**Published:** 2022-05-10

**Authors:** Dan Zhang, Yikun Ren, Yuanmeng He, Rong Chang, Shen Guo, Shanshan Ma, Fangxia Guan, Minghao Yao

**Affiliations:** School of Life Science, Zhengzhou University, 100 Science Road, Zhengzhou, 450001, PR China

**Keywords:** Gallic acid-grafted hydrogel, Reactive oxygen species-scavenging activity, *In situ* forming, Biocompatible, Traumatic brain injury

## Abstract

The efficacy of neural repair and regeneration strategies for traumatic brain injury (TBI) treatment is greatly hampered by the harsh brain lesion microenvironment including oxidative stress and hyper-inflammatory response. Functionalized hydrogel with the capability of oxidative stress suppression and neuroinflammation inhibition will greatly contribute to the repairment of TBI. Herein, antioxidant gallic acid-grafted hyaluronic acid (HGA) was combined with hyaluronic acid-tyramine (HT) polymer to develop an injectable hydrogel by dual-enzymatically crosslinking method. The resulting HT/HGA hydrogel is biocompatible and possesses effective scavenging activity against DPPH and hydroxyl radicals. Meanwhile, this hydrogel improved cell viability and reduced intracellular reactive oxygen species (ROS) production under H_2_O_2_ insult. The *in vivo* study showed that *in situ* injection of HT/HGA hydrogel significantly reduced malondialdehyde (MDA) production and increased glutathione (GSH) expression in lesion area after treatment for 3 or 21 days, which might be associated with the activation of Nrf2/HO-1 pathway. Furthermore, this hydrogel promoted the microglia polarization to M2 (Arg1) phenotype, it also decreased the level of proinflammatory factors including TNF-α and IL-6 and increased anti-inflammatory factor expression of IL-4. Finally, blood-brain barrier (BBB) was protected, neurogenesis in hippocampus was promoted, and the motor, learning and memory ability was enhanced. Therefore, this injectable, biocompatible, and antioxidant hydrogel exhibits a huge potential for treating TBI and allows us to recognize the great value of this novel biomaterial for remodeling brain structure and function.

## Graphical abstract

An injectable and biocompatible hyaluronic acid hydrogel with reactive oxygen species-scavenging activity was designed to improve traumatic brain injury treatment through suppressing oxidative stress and neuroinflammation.

**Figure:**
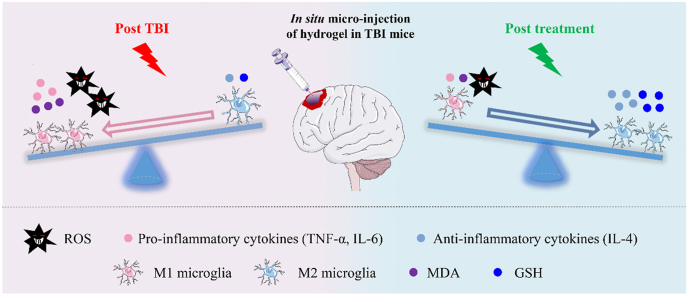

## 1 Introduction

Traumatic brain injury (TBI) is a growing devastating neurotrauma caused by mechanical force on the brain, which leads to a high mortality and disability worldwide. Even after a timely and successful treatment, most survived TBI patients still need a long-term care or rehabilitation [1,2]. And the health burden imposes significantly on families and society. It has been reported that the global cost for TBI is about $400 billion annually [3]. Therefore, there is an urgent need to find effective therapeutic strategy for neural functional repair.

The physiopathology associated with TBI mainly falls into two categories: (i) primary injury, which is directly caused by mechanical impact; and (ii) secondary injury, which refers to further tissue and cellular damage following primary insult [4]. The secondary injury is a result of several biochemical and molecular mechanisms, including neuronal excitotoxicity, oxidative stress, mitochondrial dysfunction and a hyper-inflammatory response to injury, finally leading to long-term neurological disorders [5]. Thus, focusing on secondary injury cascades will be of high practical value for clinical intervention and targeted therapies [6,7].

The era of neural regeneration therapy is revolutionary. Stem cell transplantation has shown improved neural functional recovery in more and more studies. However, the efficacy for neural repair still needs to be improved because of low cell survival, migration, retention ratio and differentiation ratio [8,9]. In addition, the harsh brain lesion microenvironment further limited the effect of stem cell-based therapy [10]. Therefore, targeting the unfavorable lesion microenvironment such as free radicals and neuroinflammation after TBI is promising for neural function recovery [11,12]. To date, lots of ROS scavengers (such as glutathione and mitochondria-targeted peptide SS-31) and anti-inflammatory drugs (such as sinomenine and salsalate) have been used for TBI treatment [[13], [14], [15], [16]]. Recently, nanozymes with ultrahigh reactive oxygen or nitrogen species selectivity have also been attempted to eliminate the free radicals and alleviate inflammation levels in the brain lesion [[17], [18], [19], [20]]. However, these above strategies are mainly based on oral, intraperitoneal, or intravenous administration routes and still face many challenges including limited diffusion through the blood-brain barrier, quickly elimination by blood circulation, and multiple dosing to ensure the long-term effect.

Hydrogels with excellent biocompatible property can be designed to mimic the extracellular matrix to provide a living habitat for cells, they are widely used in biomedicine, especially in regenerative medicine [21]. At the same time, hydrogels can be injected *in situ* to fill the defect, which have the advantages of targeted therapy, minimal trauma, high safety and easy operation. Qian group has developed a curcumin-loaded TM/PC hydrogel, which played anti-inflammatory and ROS depletion roles to promote neurogenesis after TBI [22]. Additionally, Lin group has designed an oxidized methylcellulose hydrogel for sustaining release of vitamin C, which resulted in a significant improvement in the outcome in TBI rats [23]. Natural ingredients extracted from living organism are widely used in biomedical fields due to their safety and beneficial effects. Gallic acid (GA), a low molecular tri-phenolic compound, is a secondary metabolite presented in most plants [24]. Apart from inherent biocompatibility, GA also possesses free radical scavenging ability, anticarcinogenic, antimutagenic and antimicrobial properties [[25], [26], [27]]. It has been reported that GA alleviated gouty arthritis by inhibiting NLRP3 inflammasome and enhancing Nrf2 signaling [28]. Besides, GA also weakened inflammatory response by reducing the release of inflammatory cytokines [29]. GA-grafted hydrogels have been employed for skin wound healing through regulating ROS level [30,31]. However, the therapeutic effect of GA-grafted and antioxidant hydrogel on TBI by controlling oxidative stress and neuroinflammation has not been explored yet.

**Scheme 1:**
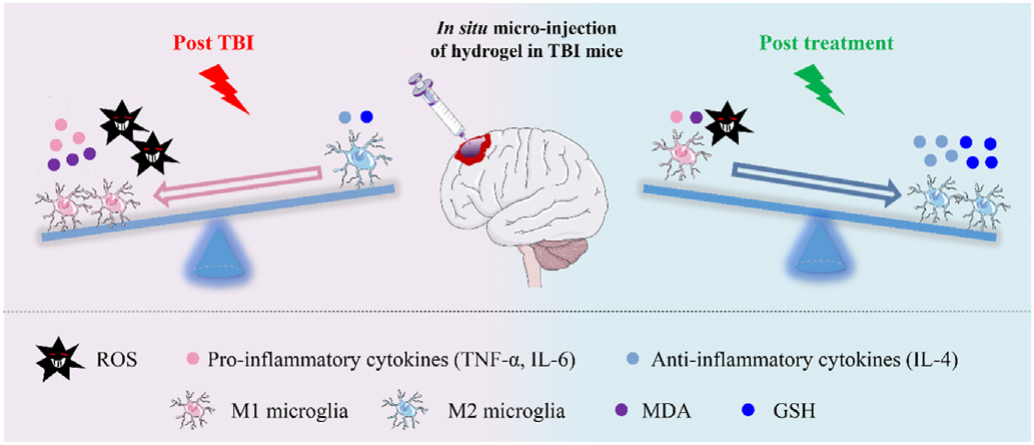
*In situ* micro-injection of HT/HGA hydrogel improved neural functional repair in TBI model by scavenging free radicals and shifting microglial polarization from a pro-inflammatory M1 phenotype to an anti-inflammatory M2 phenotype.

Therefore, in this study, we intend to design an injectable, biocompatible, and GA-grafted hydrogel with free radicals scavenging ability for TBI treatment. Recently, our group have developed a HT (hyaluronic acid grafted with tyramine) hydrogel which can be *in situ* covalently crosslinked via horseradish peroxidase (HRP) and galactose oxidase (GalOx) mediated coupling of phenol moieties on tyramine, and this hydrogel possesses excellent injectability and biocompatibility [32]. Herein, HGA conjugate (hyaluronic acid grafted with gallic acid) was fabricated and incorporated into HT hydrogel to create an injectable and biocompatible HT/HGA hydrogel with enhanced free radicals scavenging property compared to HT hydrogel alone. Through the *in situ* micro-injection approach at the brain lesion, this antioxidant hydrogel presented an obviously positive effect on the inhibition of oxidative stress and inflammatory reaction, and finally promoted the recovery of motor, learning and memory functions of TBI mice. The molecular mechanism of enhanced TBI treatment by hydrogel implantation is presented in Scheme 1. We expect that this injectable, biocompatible, and antioxidant HT/HGA hydrogel system provides a potential therapy for TBI.

## 2 Materials and methods

### 2.1 Materials

Hyaluronic acid sodium salt (HA) from bovine vitreous humor was purchased from Sangon Biotech Co., Ltd. (Shanghai, China). Gallic acid (GA), adipic acid dihydrazide (ADH), tyramine hydrochloride (Tyr), galactose oxidase (GalOx, ≥ 30 units/mg, solid), horseradish peroxidase (HRP, ≥ 160 units/mg, solid), 2-(N-morpholino)ethanesulfonic acid (MES), 1-ethyl-3-(3-dimethylaminopropyl)-carbodiimide (EDC), N-hydroxy succinimide (NHS) and DCFH-DA (2,7-dichlorodihydrofluorescein diacetate) were obtained from Aladdin Chemical Reagent Co., Ltd. (Shanghai, China). Dimethylformamide (DMF) was obtained from Sinopharm Chemical Reagent Co., Ltd (Shanghai, China). Evans blue dye, dialysis membrane (molecular cutoff ​= ​3500 ​Da), malondialdehyde (MDA), glutathione (GSH), alkaline phosphatase (ALP), aspartate transaminase (AST) and glutamic-pyruvic transaminase (GPT) assay kits were purchased from Solarbio Science & Technology Co., Ltd. (Beijing, China). IL-4, IL-6 and p-Nrf2 antibodies were purchased from Affinity Biosciences (Cincinnati, OH, USA), the remaining antibodies were sourced from Proteintech Group Inc. (Wuhan, China).

### 2.2 Synthesis and characterization of HT and HGA conjugates

The hyaluronic acid-tyramine (HT) was prepared using a previously reported method [32]. For gallic acid-conjugated hyaluronic acid (HGA), 0.3 ​g hyaluronic acid (HA) was firstly added to MES solution, and the mixture was heated to 60 ​°C until completely dissolved. Then EDC and NHS were added and stirred at room temperature for 30 ​min to activate carboxyl groups of HA, followed by adding 1 ​g ADH and stirring at room temperature for 24 ​h reaction. Finally, the solution was transferred into a dialysis bag and dialyzed against deionized water for 3 days to obtain HA-ADH solution. GA was activated by EDC and NHS in a co-solvent of water and DMF (volume ratio of 3: 2) for 60 ​min at room temperature. Then the activated GA solution was added to prepared HA-ADH solution and stirred at room temperature for 24 ​h in nitrogen atmosphere. The resulting solution was dialyzed and lyophilized to obtain HGA conjugate. The chemical structures of HT and HGA polymers were characterized by nuclear magnetic resonance (^1^H NMR). And the absorption measurements were determined by Shimadzu UV–visible spectrophotometer, grafting degrees of Tyr and GA were calculated according to standard curves.

### 2.3 Preparation and characterization of HT/HGA hydrogels

0.5% (w/v) HT and HGA (0.25%, 0.5% and 0.75%) conjugates were dissolved in 100 ​mM d-Galactose solution, followed by adding 3 U mL^−1^ GOX and 1 U ​mL^−1^ HRP solution to induce gelation for HT_0.5_, HT_0.5_HGA_0.25_, HT_0.5_HGA_0.5_ and HT_0.5_HGA_0.75_ hydrogels. The gelation time of samples was measured by inversion method. When solution stopped flowing ≥30 ​s, gelation time was recorded. Water content was measured by freeze-drying method. First, 100 ​μL hydrogels were immersed in PBS solution at 37 ​°C water bath. After 24 ​h, hydrogels were weighted as W_w_, and hydrogel weights after freeze-drying were recorded as W_d_. Water content was calculated as follows: water content (%) ​= ​[(W_w_-W_d_)/W_w_] ​× ​100. Degradation performance *in vitro* was investigated via the formula: weight remaining ratio of hydrogels (%) ​= ​W_t_/W_i_ ​× ​100, where W_t_ and W_i_ are weights of remaining hydrogels after degradation at different time points in PBS solution and weight of initial hydrogels, respectively. For enzymatic degradation, 100 ​μL hydrogels were immersed in 15 U mL^−1^ hyaluronidase solution at 37 ​°C and the hyaluronidase solution was changed every hour. Enzymatic degradation of hydrogels then was investigated using the formula: weight of hydrogels (%) ​= ​W_e_/W_o_ ​× ​100. The initial mass of hydrogels is labeled as W_o_, and the remaining mass of hydrogels after immersion is labeled as W_e_. The rheological behavior was measured on a rheometer platform (DHR-2, TA Instruments, USA) at 37 ​°C. A dynamic oscillation angular frequency ranged from 0.1 ​rad/s to 100 ​rad/s and strain was 100%. The internal morphology was investigated using scanning electron microscope (JCM-6000Plus, JEOL, Japan). Dynamic rheological measurement was performed to evaluate the shear thinning behavior of HT_0.5_HGA_0.5_ hydrogel at a shear rate of 1–100 s^−1^.

### 2.4 Evaluation of *in vitro* free radicals scavenging activity and cell protection efficiency by hydrogels insulted by H_2_O_2_

The free radicals scavenging effect of hydrogels *in vitro* was evaluated by DPPH and hydroxyl radicals scavenging assays. The DPPH radicals scavenging activity was performed according to Ref. [33] with slight modifications. 300 ​μL hydrogels were immersed in anhydrous ethanol, and then 100 ​μL DPPH solution (0.5 ​mM) was added for 1 ​h and 24 ​h in dark. Hydrogels of control group were replaced with deionized water. The absorbance at 517 ​nm was measured with a microplate analyzer. DPPH radicals scavenge activity (%) = (OD_control_-OD_hydrogel_)/OD_control_ ​× ​100. Hydroxyl radicals were produced through the Fenton reaction [31]. 600 ​μL FeSO_4_ and 500 ​μL Safranin O solution were successively added to a test tube, then 300 ​μL hydrogels were immersed for 10 ​min at room temperature. Finally, 800 ​μL ​H_2_O_2_ solution was added and incubated at 55 ​°C water bath for 60 ​min. The hydroxyl radicals scavenging ability was calculated with the following formula: hydroxyl radicals scavenging activity (%) = (OD_hydrogel_-OD_blank_)/(OD_control_-OD_blank_) ​× ​100, where OD_control_ denotes absorbance of control group (deionized water instead of hydrogels and H_2_O_2_) at 492 ​nm, and absorbance of blank group (deionized water instead of hydrogels) is labeled as OD_blank_.

The cell protective effect of hydrogels in H_2_O_2_ induced microenvironment was evaluated by CCK-8 assay. Neuronal cells including Neuro-2a (N2a) and HT22 were seeded on 96-well plates, and cultured in DMEM/F12 complete medium (containing 10% fetal bovine serum) for 24 ​h. The media were then replaced with fresh media containing 500 ​μM ​H_2_O_2_ for N2a cells and 250 ​μM ​H_2_O_2_ for HT22 ​cells to induce oxidative stress. Cells cultured in fresh media without H_2_O_2_ were considered as control group. In the hydrogel groups, cells were treated with both H_2_O_2_ and 10 ​μL hydrogels. After 24 ​h incubation, the medium was replaced by 100 ​μL CCK-8 working fluid (10:1 ​vol ratio of DMEM/F12 medium to CCK-8 solution) and incubated in cell incubator for 2 ​h without light. The absorbance at 450 ​nm was measured with a microplate analyzer. CCK-8 working fluid was set as a blank group. Cell viability was calculated using the equation: cell viability (%) = (OD_treated_-OD_blank_)/(OD_control_-OD_blank_) ​× ​100.

Intracellular ROS level in N2a and HT22 ​cells in response to H_2_O_2_ stimulation were assessed by DCFH-DA staining [34]. First, N2a cells (6 ​× ​10^4^/mL) and HT22 ​cells (4 ​× ​10^4^/mL) were inoculated in 96-well plates for 24 ​h. The media were then replaced with complete media containing 1 ​mM ​H_2_O_2_ for 3 ​h to induce intracellular ROS production. Cells cultured in complete media without H_2_O_2_ were set as normal control group. In the hydrogel groups, cells were incubated with H_2_O_2_ and 10 ​μL hydrogels. Finally, DCFH-DA working solution (10 ​μM) was added for 30 ​min incubation at dark, and fluorescence intensity and cellular morphology were observed by fluorescence microscope and white light microscope, respectively.

### 2.5 Evaluation of hydrogels biocompatibility

The cytocompatibility of hydrogels for N2a and HT22 ​cells was evaluated by CCK-8 assay. 100 ​μL pre-fabricated sterile hydrogel samples were placed in 24-well plates with 1 ​mL DMEM/F12 medium (containing 10% fetal bovine serum) in cell incubator for 24 ​h, then samples were removed and the medium were collected. N2a and HT22 ​cells were inoculated on 96-well plates for 24 ​h, then the culture medium was replaced by collected sample extracts. Subsequently, after culturing in these extracts for 1 and 2 days, the medium was replaced by 100 ​μL CCK-8 working fluid and incubated in cell incubator for 2 ​h without light. The absorbance at 450 ​nm was measured with a microplate analyzer. Cell viability was assessed by the formula: cell viability (%) = (OD_hydrogel_-OD_blank_)/(OD_control_-OD_blank_) ​× ​100.

Hemolysis test was conducted to detect blood compatibility of hydrogels before animal experiments [35]. The prepared 100 ​μL hydrogels were incubated with 1 ​mL normal saline at 37 ​°C water bath for 30 ​min, then 20 ​μL blood was added and incubated for 1 ​h. Finally, the supernatant was centrifuged and the absorbance was measured at 545 ​nm. The hemolysis rate of each group was calculated according to the formula: Hemolysis rate (%) ​= ​[(OD_T_-OD_N_)/(OD_P_-OD_N_)] ​× ​100. OD_T_, OD_P_, and OD_N_ are the absorbance values of test group, positive control group (20 ​μL blood and 1 ​mL deionized water without hydrogels) and negative control group (20 ​μL blood and 1 ​mL normal saline without hydrogels), respectively.

To evaluate the biocompatibility of HT_0.5_HGA_0.5_ hydrogel *in vivo*, it (100 ​μL) was injected under the skin of C57 mice. The surrounding tissues were stained with hematoxylin and eosin (H&E) to examine the immune response to the hydrogel on day 3, 7 and 14. For hemanalysis, serum samples of each group were harvested on day 14, alkaline phosphatase (ALP), aspartate transaminase (AST) and glutamic-pyruvic transaminase (GPT) activities were analyzed by corresponding chemical assay kits, serum sample of normal C57 mice were used as the control group.

### 2.6 Animals experiment

Male C57BL/6 mice (20 ​± ​2 ​g) were purchased from the Experimental Animal Center of Zhengzhou University. The animals were provided with diet and water for 7 days in our laboratory to acclimatize environment. All animal treatment including surgical procedures and post-operative care were conducted according to Guidelines for Care and Use of Laboratory Animals of Zhengzhou University.

#### 2.6.1 Establishment of moderate mice TBI models and treatment

TBI animal model was established using a typical Feeney's weight-drop method with slight modification [36]. Mice were deeply anesthetized with 10% chloral hydrate by intraperitoneal injection. Prior to surgery, hair above skull of mice were shaved off. An incision about 1.5 ​cm was made in the midline of scalp, and a 3 ​mm craniotomy performed between the bregma and the lambda with 1.5 ​mm lateral to the midline in the right hemisphere. The 3 ​mm section of skull cap was carefully removed so that the underlying dura was not damaged. Next, mice were fixed on stereotaxic instruments (Shenzhen Ruiwode Lift Technology Co. Ltd, China), and a craniocerebral percussion device (Shenzhen Ruiwode Lift Technology Co. Ltd, China) was adjusted so that the striker was vertical to the dura. The hammer was lowered 2 ​mm further when the striker contacted with dura, and the 20 ​g hammer was freely dropped from 20 ​cm height, resulting in moderate brain injury. The TBI mice were randomly divided into 4 groups (n ​≥ ​6 each group, multiple batches), including Sham, Normal Saline (NS), HT, and HT/HGA (HT_0.5_HGA_0.5_) groups. After cessation of bleeding, 20 ​μL HT_0.5_ pre-hydrogel solution or HT_0.5_HGA_0.5_ pre-hydrogel solution was injected into subdural mater using a 100 ​μL micro-syringe with a process lasting 1 ​min in HT group and HT/HGA group, respectively. Whereas TBI mice in NS group were treated with 20 ​μL normal saline in the same way. Sham mice that received only a craniotomy were used as control group. Finally, the scalp was closed with black silk sutures. Mice were kept warm until they became active.

#### 2.6.2 Evans blue staining

The mice were injected with 2% Evans blue solution (5 ​mL/kg) through the caudal vein on the 3rd day after treatment, as previously reported [37]. After 2 ​h, the mice were transcardiacally perfused with saline to clear the circulating Evans blue dye and sacrificed. Frozen slices of 10 ​μm thick were harvested and photographed under a fluorescence microscope.

#### 2.6.3 Detection of MDA and GSH level

To reveal the changes of lipid peroxide level and glutathione (GSH) content after treatment, we collected brain tissues around lesion area at day 3 and day 21, and used commercial kits to measure malondialdehyde (MDA) and GSH level.

#### 2.6.4 Neurological behavior assessment

Neurological behavior including motor, cognition and emotion were assessed respectively. Motor function of mice was evaluated using modified neurological severity score (mNSS), as described previously [38]. The higher score means the more severe neurological deficit. The test was carried out on day 1, 3, 7, 14 and 21.

The learning and memory abilities were evaluated with Morris water maze (MWM) test on 16–21 days post-TBI, as previously described [39]. The maze was a round pool, which was 150 ​cm in diameter and 60 ​cm in depth. The pool was filled with water and evenly divided into four quadrants (I, II, III, IV) and titanium dioxide was added into the water so that the water was white to record mice trails better. A platform with 15 ​cm diameter located 2 ​cm under water in the I quadrant of the pool. The location cruise trial lasted five days, mice were placed on the experimental platform for 20 ​s to get familiar with the environment, and then were positioned facing the wall of the pool at one of four quadrants and released to find platform. If the mouse could successfully find the platform within 60 ​s, the next quadrant test would be carried out directly. If the mouse could not find the platform within 60 ​s, it was guided to the platform and allowed to rest on it for 20 ​s again for the next quadrant test. On the sixth day, the platform was removed, each mouse was tested from IV quadrant to assess cognitive function. The movement of each mouse was recorded by a video camera above the maze and parameters, including escape latency, time in I quadrant, and number of platform-crossing, were calculated using the SLY-WMS Morris analysis system (Beijing Shuolinyuan Technology Co., Ltd).

After brain trauma, animals or patient always present psychiatric symptoms such as anxiety or depression. The sucrose preference test (SPT) was regarded as a measure of anhedonia [40]. Mice were housed individually during the SPT trial. In the first 3 days for adaptation period, two jars of sucrose water with a concentration of 1% (w/v) were provided to the mice, and then a jar of deionized water was substituted for a jar of sucrose water before formal test lasting 3 days. All bottles were weighted before and after the test, and sucrose preference index was calculated by the formula: sucrose water preference index (%) ​= ​sucrose water consumption/(sucrose water consumption ​+ ​distilled water consumption) ​× ​100.

#### 2.6.5 Western blot

After 3 days’ treatment, mice were anesthetized with 10% chloral hydrate (4 ​mL/kg intraperitoneally) and transcardially perfused with 0.9% saline. Brain tissues were harvested and damaged tissue was homogenized in protein extract solution for 30 ​min, then the homogenate was centrifuged and total protein concentration of supernatants was quantified using the BCA protein assay kit. Protein samples were separated and electro-transferred onto PVDF membranes, blocked with 8% skimmed milk at 4 ​°C for overnight and then incubated with primary antibodies against Nrf2, p-Nrf2, SOD1, HO-1, ZO-1 and β-actin at room temperature for 2 ​h. Then the membranes were washed with TBST solution three times for 10 ​min each time and incubated with secondary antibodies at room temperature for 2 ​h. Signals were detected by chemiluminescence using ECL substrate. Band density was quantified using Image J software.

After treatment for 21 days, the expression of apoptosis related proteins (Bax, Bcl2), inflammation related factors (IL-6, IL-4 and TNF-α) and neuron related protein (β-III tubulin, NeuN, NSE and NFL) were detected.

#### 2.6.6 Immunofluorescence

After treatment for 21 days, mice were anesthetized and then transcardially perfused with 0.9% normal saline followed by 4% paraformaldehyde. Brain tissue was fixed, dehydrated, and sliced with 10 ​μm thickness. The inflammatory reaction in lesion site was detected through inducible nitric oxide synthase (iNOS) and arginase 1 (Arg1) primary antibodies. And the proliferation and activity of neural cells in the hippocampus were detected using Ki67 and NeuN primary antibodies, respectively.

#### 2.6.7 In vivo toxicity analysis of vital organs

The systemic biosafety of hydrogels was assessed *in vivo*. H&E staining was applied to the vital organs (heart, liver, spleen, lung and kidney) of mice in each group on day 21 post-transplantation, normal C57 mice were set as control group.

#### 2.6.8 Timeline

**Scheme 2:**
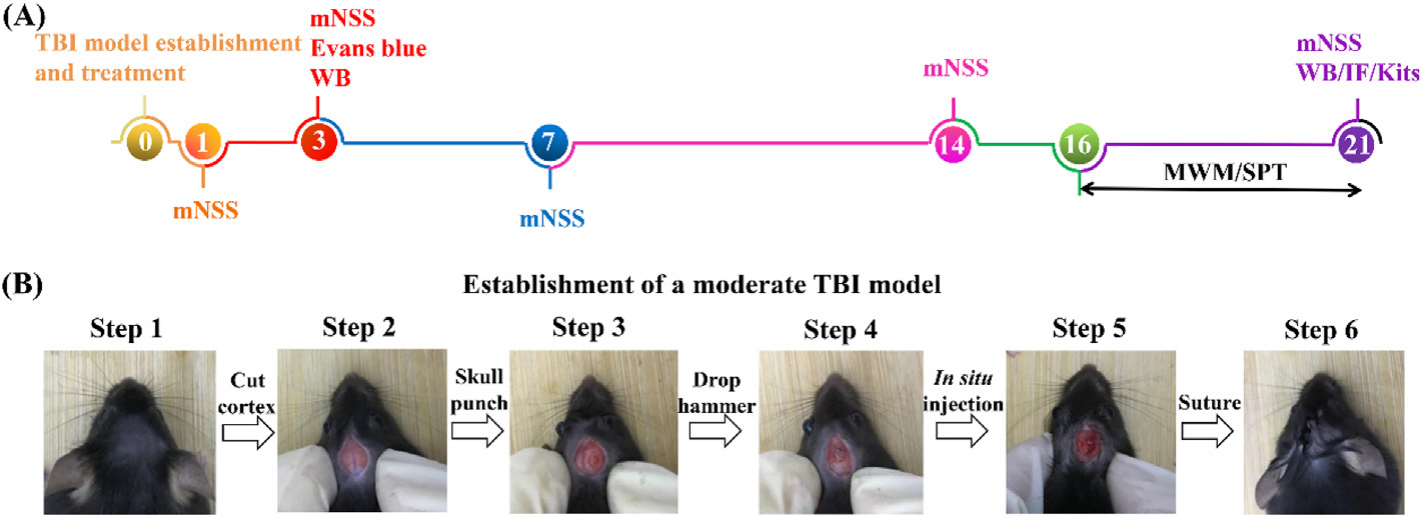
(A) Experimental timeline of procedures and (B) establishment of a moderate TBI model.

HT/HGA hydrogel was *in situ* transplanted after moderate TBI establishment (Scheme 2A-B). On the 3 days post-injury, Evans blue staining and western blot (WB) were performed to access the blood-brain barrier (BBB) integrity. To analyze the recovery of motor function, modified neurologic severity scores (mNSS) was conducted on 3, 7, 14 and 21 days after treatment. Morris water maze (MWM) test and sucrose preference test (SPT) were performed to evaluate cognitive ability and depression-like behavior of mice from day 16 to day 21. The level of malondialdehyde (MDA) and glutathione (GSH) in the injured hemisphere were examined through assay kits to observe oxidative stress after treatment for 3 and 21 days. Finally, WB and immunofluorescence (IF) staining were used to detect the expression of inflammatory factors and neurogenesis-related proteins, and H&E staining of heart, liver, spleen, lung and kidney of mice were performed to evaluate the toxicity of hydrogel to the main organs after transplantation for 21 days (day 21).

### 2.7 Statistical analysis

Data are presented as Mean ​± ​Standard deviation (Mean ​± ​SD). Statistical analysis was carried out with Graph Pad Prism 8. One-way or two-way ANOVA were used for comparisons. A statistically significant difference was considered as *p* ​< ​0.05.

## 3 Results and discussions

### 3.1 Synthesis and characterization of HT and HGA conjugates

**Fig. 1:**
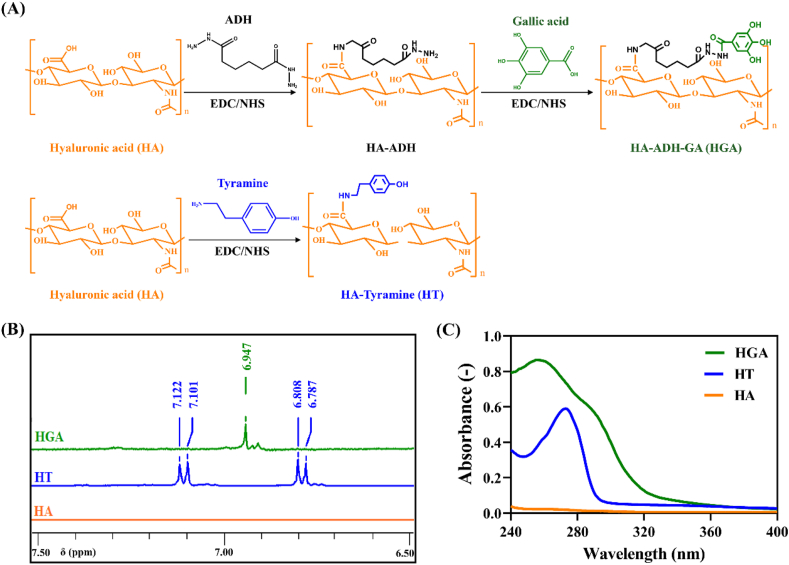
Synthesis and characterization of HT and HGA conjugates. (A) Synthetic scheme; (B) ^1^H NMR spectra and (C) UV–Vis spectra of HGA and HT conjugates.

As shown in Fig. 1A, tyramine (Tyr) and gallic acid (GA) were grafted onto hyaluronic acid (HA) macromolecular chain *via* EDC/NHS-mediated coupling reaction. Herein, we confirmed the chemical structures of hyaluronic acid-tyramine (HT) and hyaluronic acid-gallic acid (HGA) polymers through ^1^H NMR and UV–vis spectra. The obvious characteristic peak of 6.947 ​ppm in HGA conjugate was assigned to the phenyl protons of GA in ^1^H NMR spectra [41] (Fig. 1B). Phenol groups (6.7–7.1 ​ppm) of HT polymer was characterized as previously reported [42]. Compared with unmodified HA, the UV–vis spectrum of HGA conjugate showed a new peak at 256 ​nm belonging to the aromatic hydrocarbons of GA molecules [43], HT polymer presented UV absorbance at 275 ​nm corresponding to the absorbance of Tyr [32] (Fig. 1C). Taken together, these results confirmed the successful conjugation of Tyr and GA to HA chain. Calibrated by standard curves (Figure S1) and UV–vis spectra analysis, the substitution degree of Tyr and GA were determined to be 44.37 ​μmol/g of HT conjugate and 104.6 ​μmol/g of HGA conjugate, respectively.

### 3.2 Preparation and characterization of HT/HGA hydrogels

**Fig. 2:**
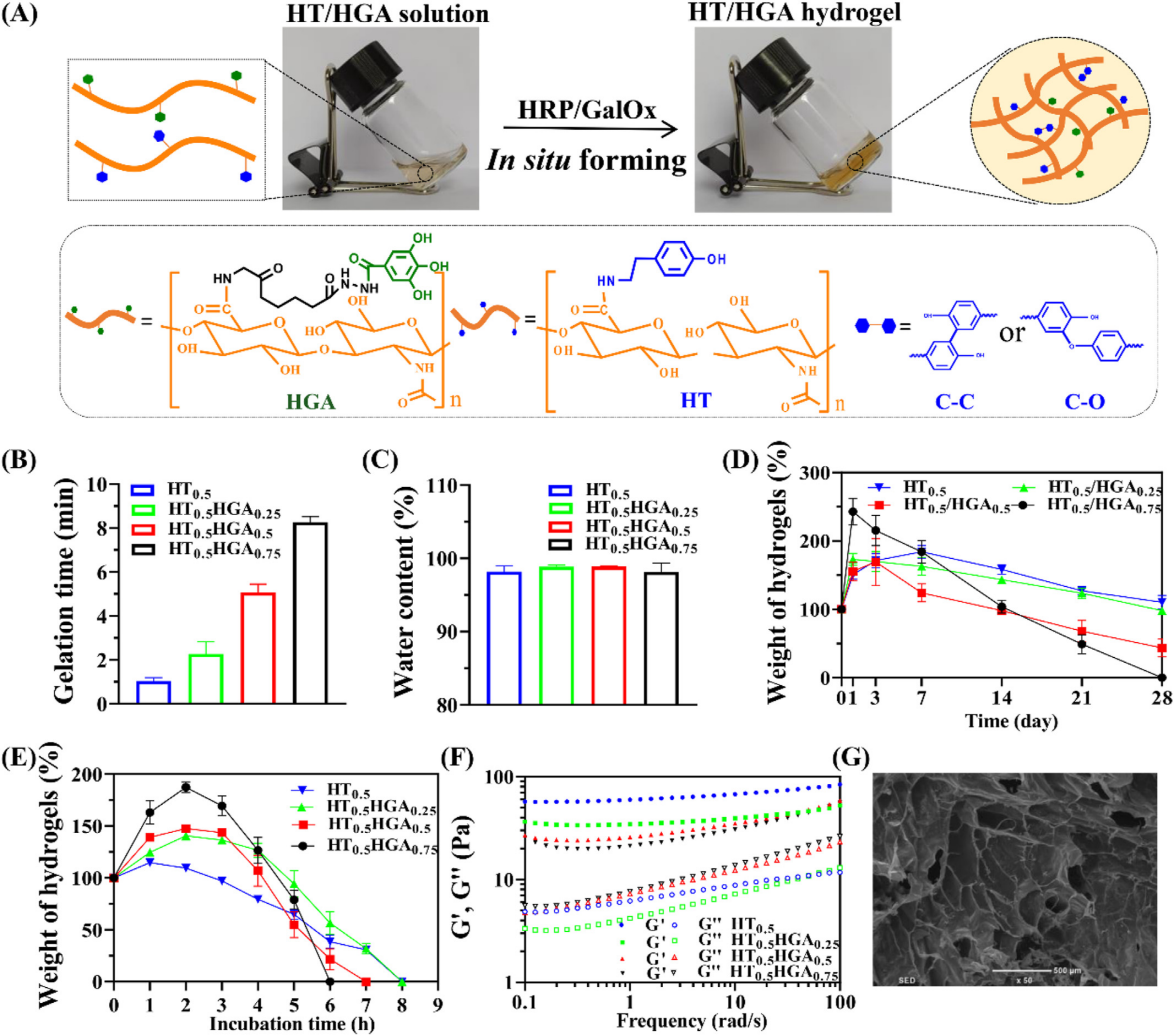
Preparation and characterization of HT/HGA hydrogels. (A) *In situ* forming hydrogels cross-linked by HRP and GalOx; (B) gelation time; (C) water content; (D) degradation in PBS solution; (E) enzymatic degradation; (F) rheology property of HT/HGA hydrogels; (G) internal microtopography of HT_0.5_HGA_0.5_ hydrogel, scale bar represented 500 ​μm.

HT/HGA hydrogels were formed *in situ* by dual-enzymatically catalyzed crosslinking of phenol-rich polymers (Fig. 2A). In the presence of horseradish peroxidase (HRP) and H_2_O_2_ generated from the oxidase of d-galactose catalyzed by galactose oxidase (GalOx), cross-linking reaction occurred through either C–C bonds between ortho-carbons of the aromatic ring or through C–O bonds between ortho-carbons and phenolic oxygen. Controllable gelation time is an important parameter for hydrogels which affects their application. According to our previous report [32], the gelation time was related to the concentration of GalOx solution and HT polymer. In this study, different content of HGA conjugates were introduced to HT polymer with 1 U mL^−1^ HRP and 3 U mL^−1^ GalOx concentration to study whether the addition of HGA affected gelation time. As shown in Fig. 2B, HT_0.5_ hydrogel was gelled within 60 ​s, and the gelation became slow with the increase of HGA content. The result may be caused by the removal of H_2_O_2_ (generated from the oxidation of d-galactose by GalOx) by HGA conjugate in the crosslinking reaction, which indicated strong scavenging effect of HGA polymer against H_2_O_2_. The gelation time was 5.07 ​± ​0.38 ​min for HT_0.5_HGA_0.5_ hydrogel. Results from Fig. 2C showed that all samples exhibited high water content exceeding 90%, which was similar with natural extracellular matrix and capable to minimize the frictional irritation between hydrogels and brain tissue. Considering the application in biomedical field, the degradation performance of hydrogels was tested. PBS solution (pH ​= ​7.4) at 37 ​°C *in vitro* were adopted to imitated the normal physiological microenvironment. As shown in Fig. 2D, all samples showed stability over 21 days, and incorporation of HGA accelerated the degradation rate of hydrogels. And they all gradually lost their mass in hyaluronidase solution within 8 ​h (Fig. 2E), indicating that these hydrogels exhibited desirable degradation characteristic for *in vivo* application. Storage modulus (G′) and loss modulus (G″) of hydrogels were investigated by rheological study (Fig. 2F). G′ values gradually increased with the increase of frequency and G′ values were obviously greater than G″ values in all samples, indicating solidified-like behavior. Furthermore, G′ values of these four hydrogel groups were all within G′ range of brain tissue (30–3000 ​Pa) when frequency was 100 ​rad/s [44], suggesting that they could be used as neural scaffolds. The shear-thinning property of HT_0.5_HGA_0.5_ hydrogel was presented in Figure S2, viscosity decreased with the increase of shear rate, and the hydrogel could be injected into the shape of ‘SCI’ letters, demonstrating the well injectability of this hydrogel. Finally, the morphology of HT_0.5_HGA_0.5_ hydrogel was observed by scanning electron microscope (SEM) and showed in Fig. 2G. It presented in porous and interconnected structures, and the pore size was 346.00 ​± ​36.39 ​μm which would facilitate the exchange of oxygen and nutrients.

### 3.3 Antioxidant performance of HT/HGA hydrogels *in vitro*

Oxidative stress induced by overproduction and accumulation of free radicals destroyed cell structure and function. These radicals attacked lipids, proteins, and DNA macromolecule polymers which resulted in cell dysfunction or apoptosis [45]. Therefore, hydrogels with free radicals scavenging ability would benefit TBI treatment by preventing these oxidative stress injuries. It has been reported that GA possessed excellent antioxidant property to scavenge free radicals, protect neural cells against cell death and morphological changes [46]. Herein, we investigated antioxidant activity of HT/HGA hydrogels by assessing the scavenging efficiency on DPPH and hydroxyl radicals *in vitro* and the effect of hydrogels on cell viability and intracellular reactive oxygen species (ROS) production in response to H_2_O_2_.

**Fig. 3:**
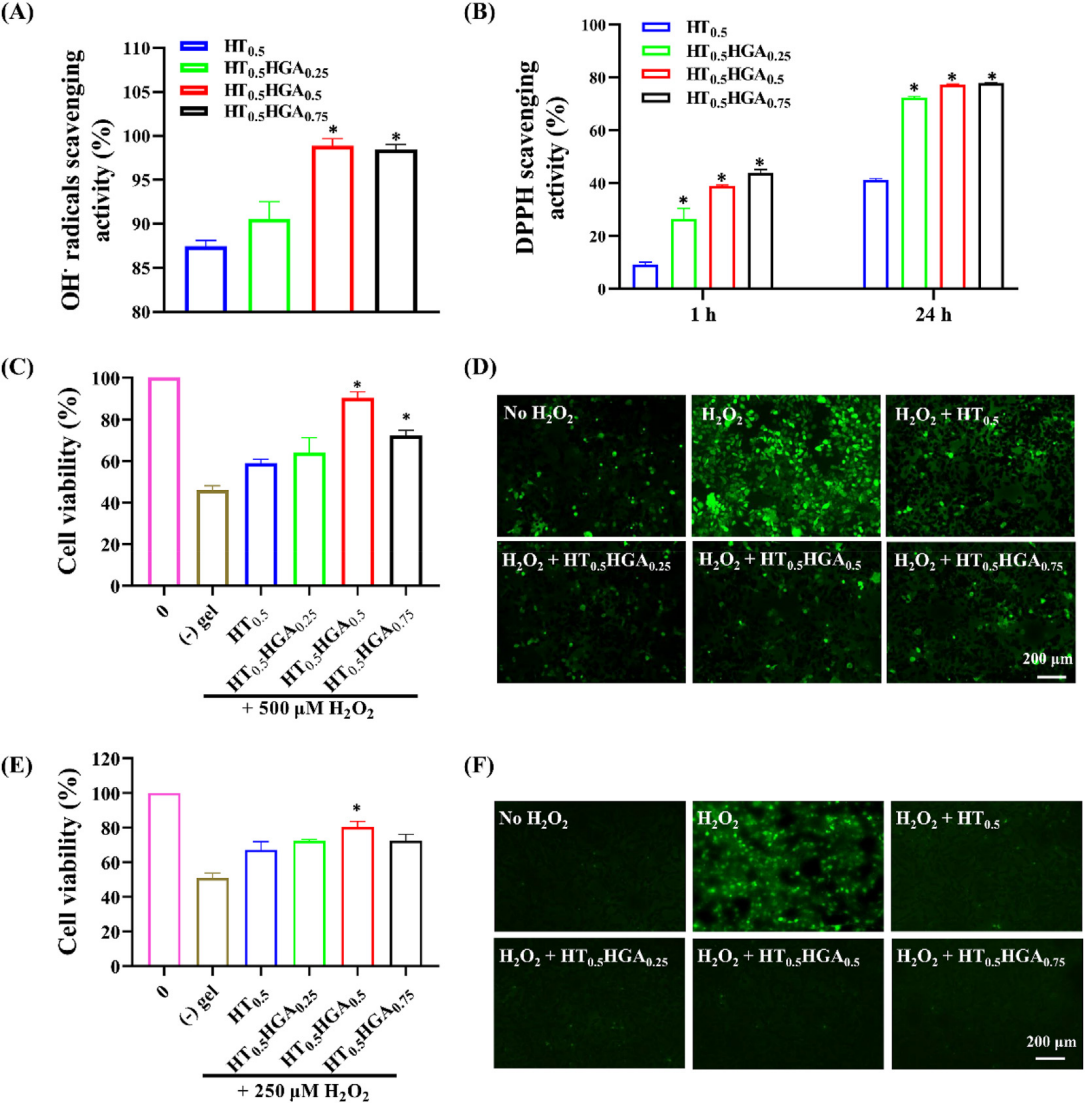
Antioxidant performance of HT/HGA hydrogels *in vitro*. (A) Hydroxyl radicals scavenging ability of hydrogels for 1 ​h; (B) DPPH radicals scavenging ability for 1 ​h and 24 ​h; cell viability of (C) N2a and (E) HT22 with or without hydrogels in H_2_O_2_ microenvironment for 24 ​h; DCFH-DA staining for ROS detection in (D) N2a and (F) HT22 with or without hydrogels in H_2_O_2_ microenvironment for 3 ​h (∗*p*＜0.05, compared with HT_0.5_ hydrogel group, Mean ​± ​SD, n ​= ​3).

Fig. 3A showed that HT_0.5_ hydrogel could effectively scavenge 87.46 ​± ​0.25% hydroxyl radicals, perhaps because the hydroxyl groups, amine groups, and phenolic compounds on HT conjugate acted as electron donors and reacted with hydroxyl radicals. However, the HT_0.5_ hydrogel's scavenging effect against DPPH radicals was much lower, around 9% (Fig. 3B). After introduction of HGA conjugate, the scavenging ability of HT_0.5_HGA_0.5_ hydrogel against hydroxyl radicals raised to 98.89 ​± ​0.80%, which was significantly higher than that of HT hydrogel. In addition, HT/HGA hydrogels also presented greater DPPH radicals scavenging effect compared with HT hydrogel for 1 ​h and 24 ​h (*p* ​< ​0.05). The DPPH radicals scavenging ability for HT_0.5_HGA_0.5_ hydrogel could reach 77.21 ​± ​0.26% after 24 ​h incubation.

The effect of hydrogel on cell viability and intracellular ROS level in response to H_2_O_2_ were evaluated by CCK-8 assay and DCFH-DA staining, respectively. As shown in Fig. 3C, cell viability of N2a cells dramatically declined to 45.94 ​± ​2.08% when cells were induced with 500 ​μM ​H_2_O_2_ for 24 ​h, while it was significantly increased when co-cultured with HT_0.5_HGA_0.5_ hydrogel, the cell viability preserved at 90.35 ​± ​2.87%. The incorporation of antioxidant HGA conjugate protected cells against H_2_O_2_ injury by increasing ROS scavenging. Interestingly, compared with HT_0.5_HGA_0.5_ hydrogel, HT_0.5_HGA_0.75_ hydrogel-treated cells displayed reduced cell survival, which might be related to lower cytocompatibility of HT_0.5_HGA_0.75_ hydrogel. HT_0.5_ hydrogel also reduced cell death to a certain extent, which was consistent with hydroxyl radicals scavenging effect of HT_0.5_ hydrogel. Furthermore, we detected the production of intracellular ROS in N2a cells by DCFH-DA staining. The DCFH-DA is decomposed by cellular esterase and produces non-fluorescent DCFH which is subsequently oxidized by intracellular ROS to form fluorescent DCF. More ROS in cells result in more DCF and stronger fluorescence intensity, so fluorescence intensity of DCF is an indicator for the level of intracellular ROS. From Fig. 3D, N2a cells showed a stronger green fluorescence when exposed with 1 ​mM ​H_2_O_2_ for 3 ​h, indicating that a large production of ROS in cells. Meanwhile, remarked morphological changes like cell shrinkage and debris were observed in H_2_O_2_ group (Figure S3A). While decreased intensity of fluorescence was revealed when N2a cells were exposed to H_2_O_2_ in the presence of hydrogels. As shown in Fig. 3E, 250 ​μM ​H_2_O_2_ for 24 ​h declined HT22 ​cell viability to 50.94 ​± ​2.28%, and H_2_O_2_-induced cytotoxicity was attenuated in all hydrogel treated groups by CCK-8 assay. Among them, the cell viability of HT_0.5_HGA_0.5_ hydrogel treatment group showed the highest protection (80.37 ​± ​3.14%), which was significantly better than HT_0.5_ hydrogel (67.06 ​± ​4.77%) (*p* ​< ​0.05). The results of DCFH-DA probe assay (Fig. 3F) were consistent with CCK-8 assay. A strong green fluorescence and morphological changes were found when HT22 ​cells were cultured with 1 ​mM ​H_2_O_2_ for 3 ​h (Figure S3B). The fluorescence densities in cells treated with hydrogels all decreased and cell morphology remained the same as cells in control group. In consideration of both free radicals scavenging activity and cellular protection ability against H_2_O_2_-induced oxidative stress, HT_0.5_HGA_0.5_ hydrogel was finally selected for subsequent animal experiments.

### 3.4 Evaluation of hydrogels biocompatibility

**Fig. 4:**
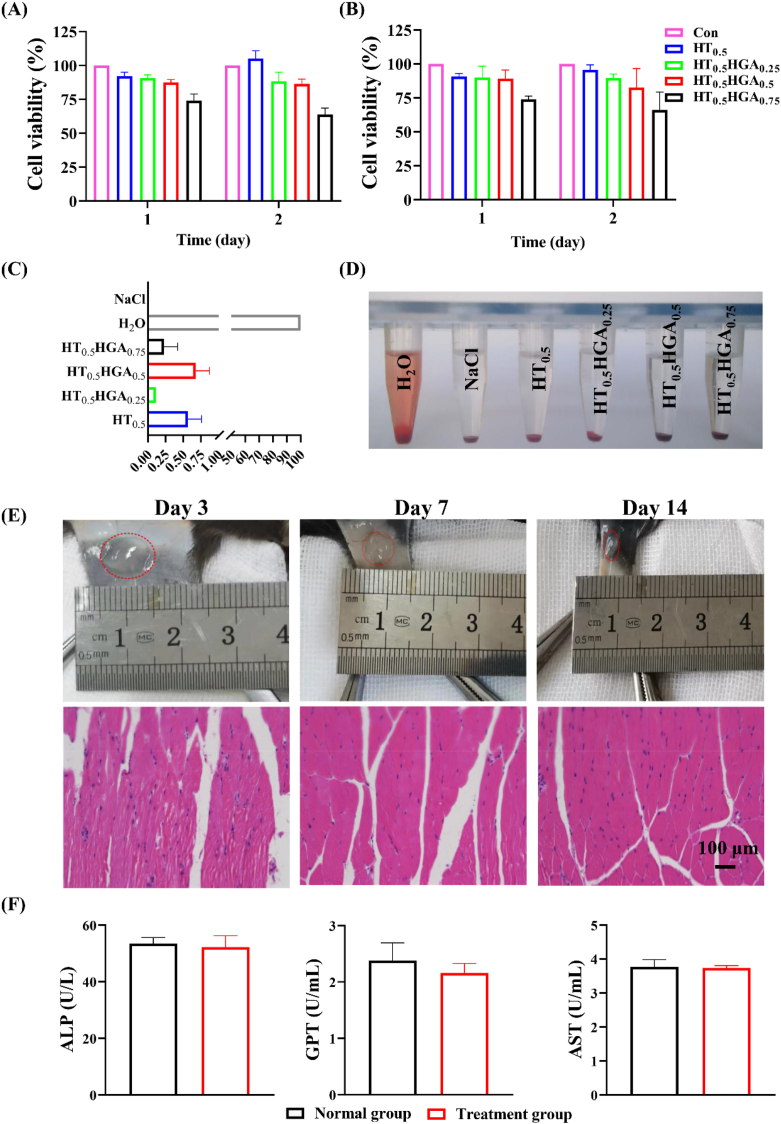
Evaluation of biocompatibility of hydrogels. The cytocompatibility of hydrogels with (A) N2a and (B) HT22 ​cells detected by CCK8 test; (C) hemolysis ratio and (D) photographs of hemolytic assay; (E) H&E staining of the surrounding tissue on day 3, 7, and 14; (F) blood biochemistry tests of ALP, GPT and AST activities in normal C57 mice and hydrogel-implanted C57 mice. Mean ​± ​SD, n ​= ​3.

Excellent cytocompatibility is the prerequisite for well-designed materials in biomedical application. CCK-8 assay was used to evaluate the cytocompatibility of HT/HGA hydrogels. For N2a (Fig. 4A) and HT22 ​cells (Fig. 4B), compared with other hydrogel groups, HT_0.5_HGA_0.75_ hydrogel showed the lowest cell viability at the first and second day. The other hydrogel formulations exhibited superior cell viability exceeding 80%. We further evaluated hemolysis ratio of these hydrogels, as shown in Fig. 4C and D, all values were far less than 5%, meeting requirement of international standards for biological materials (ISO 10993–4). Before *in vivo* application, tissue compatibility of HT_0.5_HGA_0.5_ hydrogel was evaluated by subcutaneous injection (Fig. 4E). There was no redness around injection sites. And further H&E staining indicated that this hydrogel did not induce inflammatory reaction on day 3, 7 and 14. Blood biochemistry tests were also performed to determine the liver toxicity of HT_0.5_HGA_0.5_ hydrogel. Compared with normal C57 mice in control group, there was no significant difference in serum alkaline phosphatase (ALP), aspartate transaminase (AST) and glutamic-pyruvic transaminase (GPT) activities in the hydrogel-implanted group on day 14 (Fig. 4F). These data suggested the high biocompatibility of HT_0.5_HGA_0.5_ hydrogel both *in vitro* and *in vivo*. And the hydrogel degraded gradually over time, demonstrating its biodegradability (Figure S4).

### 3.5 HT/HGA hydrogel implantation reduced the generation of MDA and increased the level of GSH through activation of Nrf2/HO-1 signal pathway

**Fig. 5:**
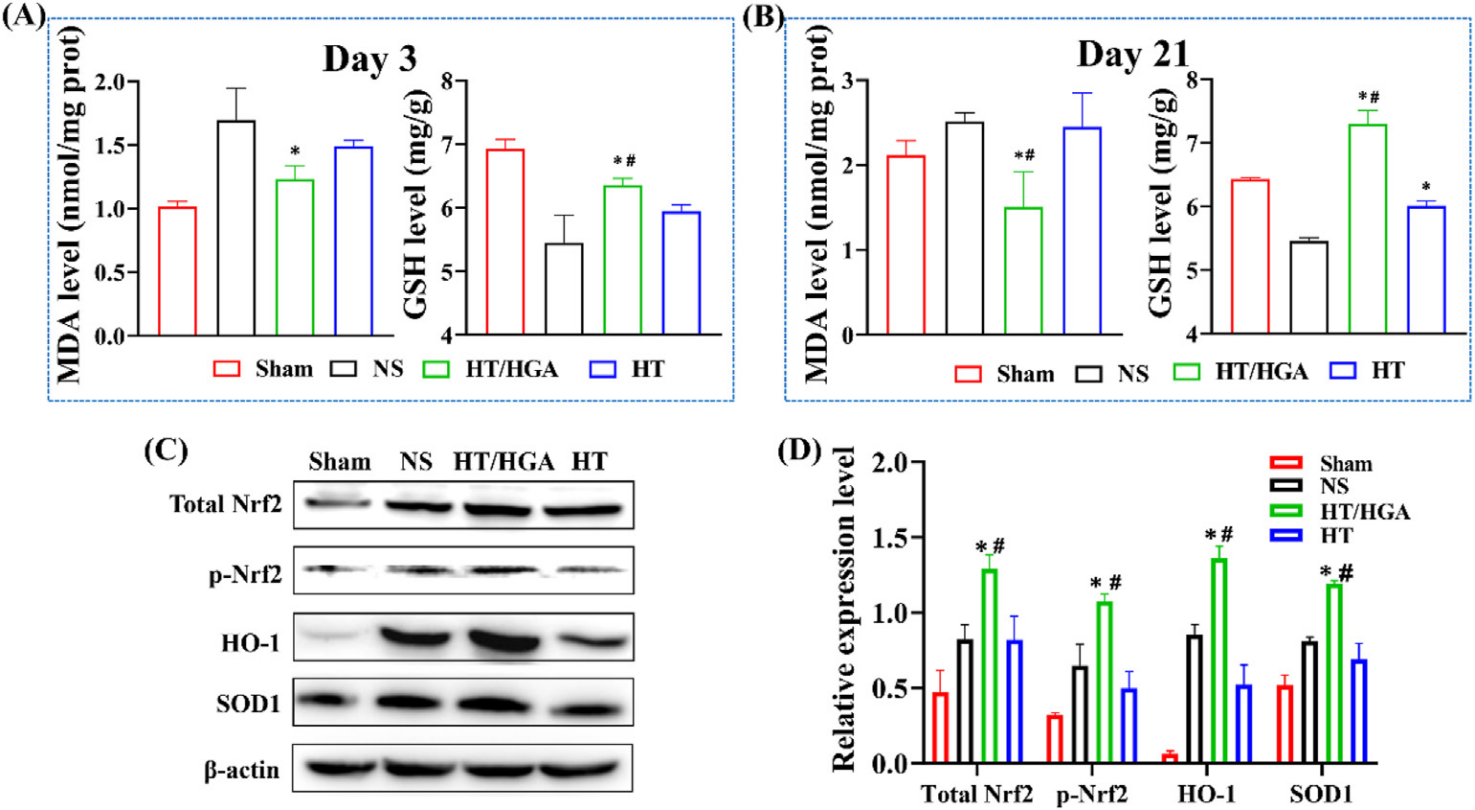
The expression level of MDA and GSH, and relevant proteins in Nrf2/HO-1 signal pathway. The expression level of MDA and GSH on day 3 (A) and day 21 (B) post-treatment; (C) western blot and (D) relative expression of total Nrf2, p-Nrf2, HO-1 and SOD1. (∗*p*＜0.05, compared with NS group; ^#^*p*＜0.05, compared with HT hydrogel group, Mean ​± ​SD, n ​= ​3).

Following TBI, scarcity of glucose and oxygen gives rise to glutamate excitotoxicity and excessive influx of calcium which promote the production of free radicals, followed by oxidative stress [47]. Oxidative stress is caused by the imbalance between generation and consumption of free radicals [48]. Polyunsaturated fatty acids are main compositions of neuronal membranes. Free radicals can aggressively react with the lipids resulting in lipid peroxidation, which ultimately destroys the integrity of blood-brain barrier (BBB) and function of the central nervous system [49]. Malondialdehyde (MDA) is one of the most important end-products of polyunsaturated fatty acid peroxidation and often regarded as an indicator of oxidative stress. Glutathione (GSH) is the most abundant low molecular weight non-protein thiol which presents in all mammalian tissues and modulates physiological levels of ROS non-enzymatically, particularly singlet oxygen and hydroxyl radicals [50]. It has been reported that GSH depletion further initiated subsequent cascade signaling which resulted in neuronal death and dysfunction [51]. To explore the effects of HT/HGA hydrogel on oxidative stress after TBI, the levels of MDA and GSH in injured hemispheres were detected on day 3 and 21 with and without hydrogel treatment. Results from Fig. 5A and B showed that HT/HGA hydrogel transplantation significantly reduced the level of MDA when compared to NS group at day 3 and 21, and there was a significant difference between the HT group and HT/HGA at day 21. Besides, HT hydrogel and HT/HGA hydrogel treatment both increased the expression level of GSH in lesion area compared with NS group at different time points, and it was higher in HT/HGA hydrogel group than HT hydrogel group (*p* ​< ​0.05). These results confirmed the positive effect of HT/HGA hydrogel on alleviating oxidative stress *in vivo*.

Nuclear factor erythroid 2-related factor 2 (Nrf2) is considered as a master regulator of detoxification, antioxidant, anti-inflammatory and cytoprotective mechanisms in the body [28]. Under physiological conditions, Nrf2 binds with kelch-like ECH-associated protein 1 (Keap1), and anchors in the cytoplasm, thus Nrf2 activity is suppressed by Keap1. While upon oxidative stress, Nrf2 is usually activated by phosphorylation to dissociate from Keap1 and enters nucleus. It combines with antioxidant response element (ARE) to induce activation of antioxidant stress/detoxifying enzymes and proteins such as heme oxygenase-1 (HO-1), catalase (CAT) and superoxide dismutase 1 (SOD1), which are positive against oxidative stress and damage. A previous study has proved that GA induces the activation and nuclear translocation of Nrf2 in a dose-dependent manner by competitively binding Keap1 and disturbing protein-protein interaction between Keap1 and Nrf2 [52]. Ginnalin A, another polyphenol from red maple, has also been shown to competitively bind Keap1 to activate Nrf2-regulated antioxidant defense system [53]. Herein, we evaluate whether the Nrf2/HO-1 signaling pathway is related to antioxidant activity of HT/HGA hydrogel. As indicated in Fig. 5C and D, because the brain was in an oxidative stress state following TBI, total Nrf2 level was increased and phosphorylation of Nrf2 was significantly upregulated. The expression of Nrf2 and p-Nrf2 was markedly increased in mice brain treated with HT/HGA hydrogel for 3 days compared with NS and HT groups. In addition, the expression of HO-1, a representative downstream regulator of Nrf2, was up-regulated by TBI (*p* ​< ​0.05), while its expression was markedly enhanced by HT/HGA hydrogel. We also examined the expression of SOD1, another key downstream molecule, it was a little bit higher in mice treated with saline compared with Sham group, however, it was significantly upregulated by HT/HGA hydrogel treatment. These results indicated that HT/HGA hydrogel could increase total Nrf2 level and stimulate Nrf2 phosphorylation to promote nuclear translocation, its binding to ARE triggered the downstream antioxidant proteins, such as HO-1 and SOD1 (*p* ​< ​0.05), thus providing a protective role against internal and external harmful stimuli following TBI.

### 3.6 HT/HGA hydrogel protected blood-brain barrier integrity in TBI mice

**Fig. 6:**
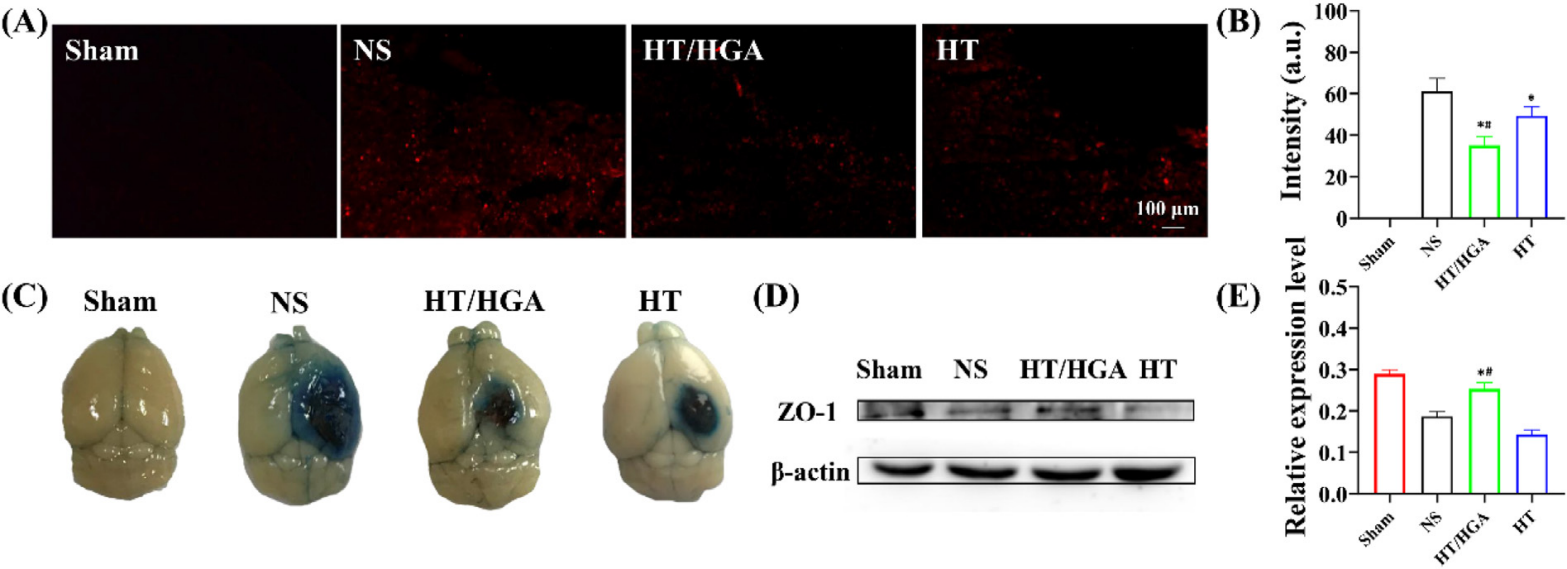
BBB permeability assessment by Evans blue staining and western blot after hydrogel treatment 3 days. (A) Fluorescence images and (B) fluorescence intensity analysis of Evans blue-labeled brain slices; (C) macroscopic Evans blue dye extravasation; (D) western blot and (E) relative expression of ZO-1. (∗*p*＜0.05, compared with NS group; ^*#*^*p*＜0.05, compared with HT hydrogel group, Mean ​± ​SD, n ​= ​3).

Blood-brain barrier (BBB) is a physical and biochemical barrier which participates in the regulation of CNS homeostasis and protects the neural tissue from toxins and pathogens [54]. It has been reported that peroxynitrite derived from superoxide and NO would aggravate BBB injury [55]. And ROS contributes to the downregulation of tight junction proteins and the activation of matrix metalloproteinases, leading to basal membrane degradation and BBB disruption [56,57]. ROS also facilitates the death of brain capillary endothelial cells by inhibiting Orai1-mediated Ca^2+^ inflow, thereby enhancing BBB leakiness [58]. It has been reported that acute administration of anti-ICAM-1/catalase preserved BBB integrity following CCI-TBI [11,59]. Hence, we speculated that HT/HGA hydrogel might maintain BBB integrity by ROS scavenging. In this study, Evans blue dye was used to assess BBB permeability on day 3 post-implantation. If BBB was damaged, Evans blue would bind to plasma albumin and enter the central nervous system. Results from Fig. 6A and B showed that the fluorescence intensity of Evans blue dramatically decreased after hydrogels treatment, compared with NS group (*p* ​< ​0.05), and the difference between HT/HGA hydrogel and HT hydrogel treatment was statistically significant (*p* ​< ​0.05). This was consistent with the result of Evans blue dye extravasation (Fig. 6C). Furthermore, to verify whether HT/HGA hydrogel could protect BBB integrity, the expression of zona occludens-1 (ZO-1) was detected by western blot. ZO-1 is one of the important protein components that make up the tight junction of BBB. As shown in Fig. 6D and E, compared with NS and HT groups, the injection of HT/HGA hydrogel markedly upregulated ZO-1 expression. In short, HT/HGA hydrogel with ROS scavenging activity could also alleviate BBB leakiness and protect BBB integrity.

### 3.7 HT/HGA hydrogel mitigated neuroinflammation of TBI model by inhibiting proinflammatory factors and promoting microglia polarization in TBI mice

Oxidative stress and inflammation are highly related processes. Oxidative stress produces and accumulates a large amount of ROS, which has been shown to accelerate the release of inflammatory signal molecules such as peroxiredoxin 2. In addition, ROS modulated synaptic and non-synaptic communication between neurons, leading to neuroinflammation [60]. Glial cells and peripheral immune cells were recruited to lesion site post-TBI, and followed by the activation of intracellular signaling pathways which amplified the inflammatory response by increasing the production of ROS [61]. Therefore, inhibition of excessive ROS production may downregulate inflammatory response.

**Fig. 7:**
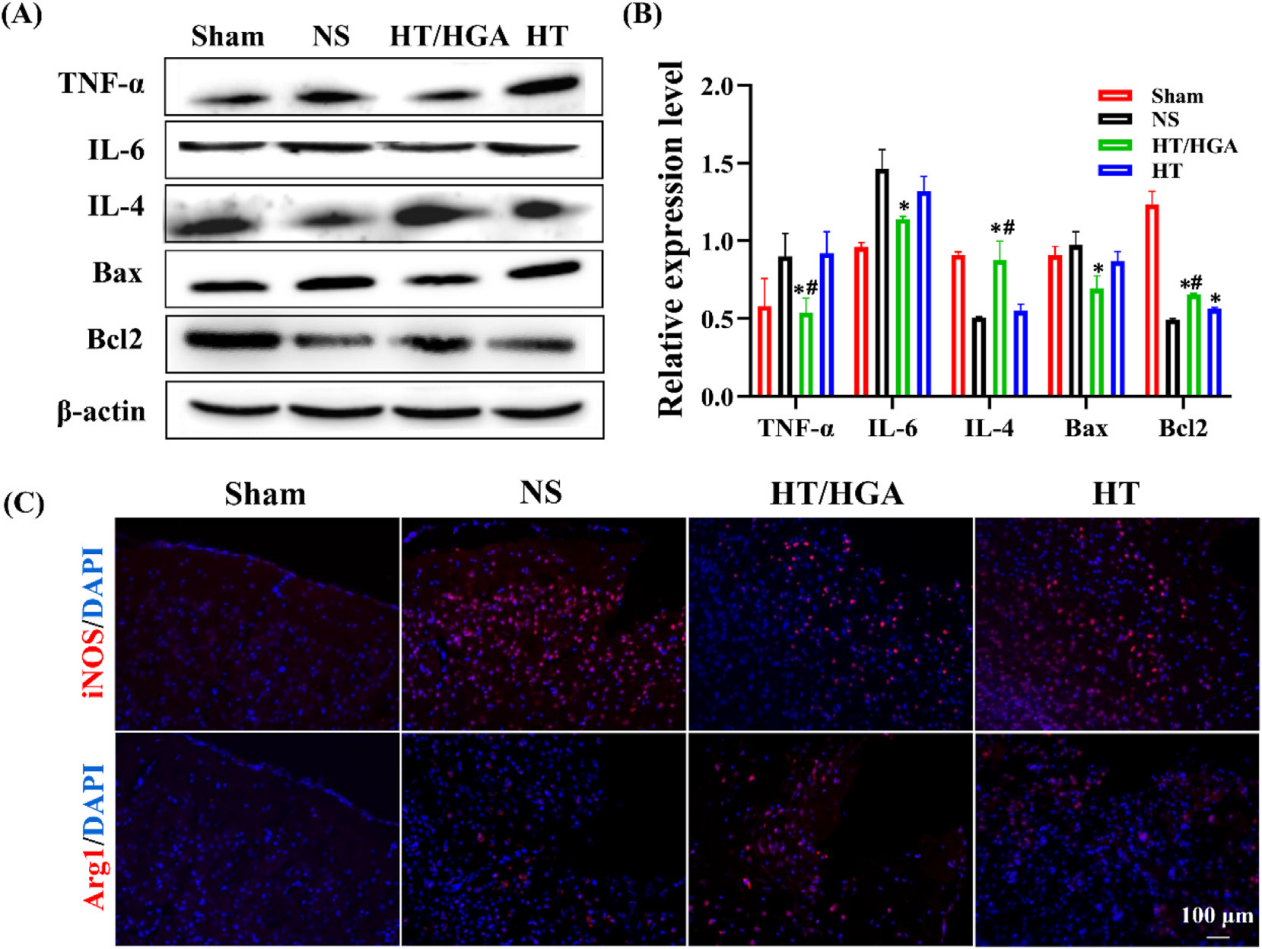
Representative inflammatory factors change and neural apoptosis proteins expression detected by Western blot and immunofluorescence staining. (A) Western blot and (B) relative expression of TNF-α, IL-6, IL-4 and Bax, Bcl2; (C) immunofluorescence staining of iNOS and Arg 1. (∗*p*＜0.05, compared with NS group; ^#^*p*＜0.05, compared with HT hydrogel group, Mean ​± ​SD, n ​= ​3).

ROS could upregulate the proinflammatory gene expression in microglia and promote the production of cytokines such as TNF-α, IL-6 and IL-1β, which have been documented to play a critical role in the inflammatory process [62,63]. To determine whether hydrogel treatment inhibited neuroinflammation, western blot analysis was used to measure the protein level of TNF-α and IL-6 in brain tissues after treatment for 21 days. The results from Fig. 7A and B showed that, mice brain in HT group presented lower expression of IL-6 without apparent difference and got significantly decreased in HT/HGA hydrogel group (*p* ​< ​0.05), compared to NS group. The expression of TNF-α was significantly downregulated in HT/HGA group compared with HT and NS groups. In addition, the expression of IL-4 in HT/HGA hydrogel group were much higher than those in NS or HT hydrogel group (*p* ​< ​0.05). When TBI occurred, oxidative injury could activate inherent apoptosis pathway to upregulate the expression of the apoptotic activator Bax and downregulate the level of anti-apoptotic factor Bcl2. There was no significant difference of Bax expression between NS group and HT group, whereas HT/HGA group displayed a lower expression compared with NS group (*p* ​< ​0.05) and HT group, indicating an enhanced suppression of neural cell apoptosis after treatment. For Bcl2, mice treated with HT and HT/HGA hydrogels displayed evidently higher expression when compared with NS group, and there was a marked difference between HT and HT/HGA hydrogel groups.

High level of inflammatory cytokines such as iNOS, IL-6, TNF-α etc., they are closely associated with inflammatory response after TBI. Microglia are resident immune cells in brain that act as the first line of defense against injury or disease in central nervous system. The M2 phenotype induces anti-inflammatory cytokines (IL-4, CD206, Arg1 etc.) release, and correlates with neuroprotective effects [64]. HT/HGA hydrogel obviously downregulated the expressions of proinflammatory cytokines (TNF-*α* and IL-6) and significantly upregulated the expression of cytokines related with reparative M2 microglia (IL-4), respectively. Immunofluorescence staining was subsequently performed to track the expression of iNOS (M1 microglia biomarker) and Arg1 (M2 microglia biomarker) after treatment for 21 days. As shown in Fig. 7C, iNOS fluorescence intensity in the lesion of TBI mice only treated with NS was obvious higher, while there were fewer Arg1-positive cells, indicating the status of microglia was mainly M1 phenotype. Evidently, HT/HGA hydrogel decreased the signal of iNOS and increased the expression of Arg1, implying this hydrogel induced the polarization from M1 to M2 microglia. The M2/M1 ratio (Arg1/iNOS) was presented in Figure S5. Taken together, HT/HGA hydrogel was proved to induce microglia polarization from M1 to M2 phenotype, which indicates the brain repair and plasticity after treatment for 21 days.

### 3.8 HT/HGA hydrogel promoted the neural function repair and alleviated depression

**Fig. 8:**
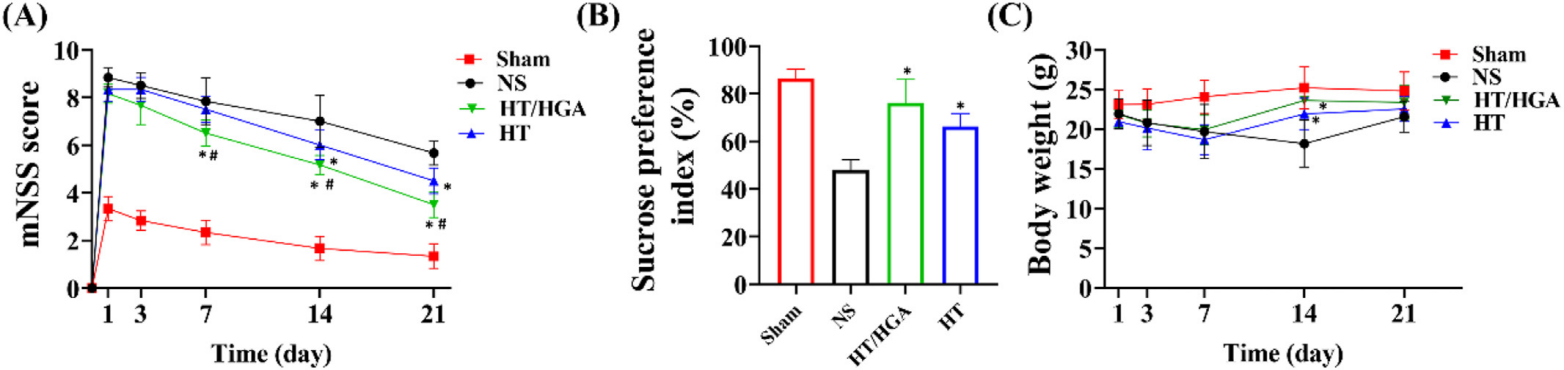
mNSS and SPT to analyze the recovery of motor ability and the depression-like behavior of TBI mice after treatment. (A) mNSS score of TBI mice on day 1, 3, 7, 14 and 21; (B) sucrose preference index of TBI mice from day 19–21; (C) body weight of TBI mice on day 1, 3, 7, 14 and 21; (∗*p*＜0.05, compared with NS group; ^#^*p*＜0.05, compared with HT hydrogel group, Mean ​± ​SD, n ​= ​6).

TBI can cause extensive sensorimotor dysfunction. To assess the neurological motor recovery after treatment, modified neurological severity score (mNSS) was performed. This test includes motor, sensory, balance and reflex reactions, the total score is 18. The higher score is positively correlated with the neurological severity [65]. As shown in Fig. 8A, scores in all groups gradually decreased over time, which indicated the motor ability of TBI mice progressively recovered. Compared with NS group, the score in HT group significantly decreased on day 14 and 21 post-treatment. The score in HT/HGA group at day 7, 14 and 21 was significantly lower than HT and NS groups, indicating HT/HGA hydrogel treatment strikingly promoted recovery of motor function.

To determine whether the hydrogel could alleviate depression-like behavior of TBI mice, sucrose preference test (SPT) was conducted (Fig. 8B). Decreased sucrose preference indicates anhedonia, which is a major symptom of depression. The sucrose consumption ratio in NS group was obviously lower than that in Sham group, demonstrating the occurrence of depression after TBI. After hydrogels treatment, decrease of the percentage of sucrose preference were reversed (*p* ​< ​0.05). And HT/HGA group presented the highest indicator of sucrose preference. In terms of body weight in each group (Fig. 8C), mice in NS group gradually lost their body weight from day 1–14 and gained weight at day 21, while mice in hydrogel groups increased weight from day 7 and were significantly different from those in NS group.

### 3.9 HT/HGA hydrogel promoted the learning and memory ability of TBI mice

**Fig. 9:**
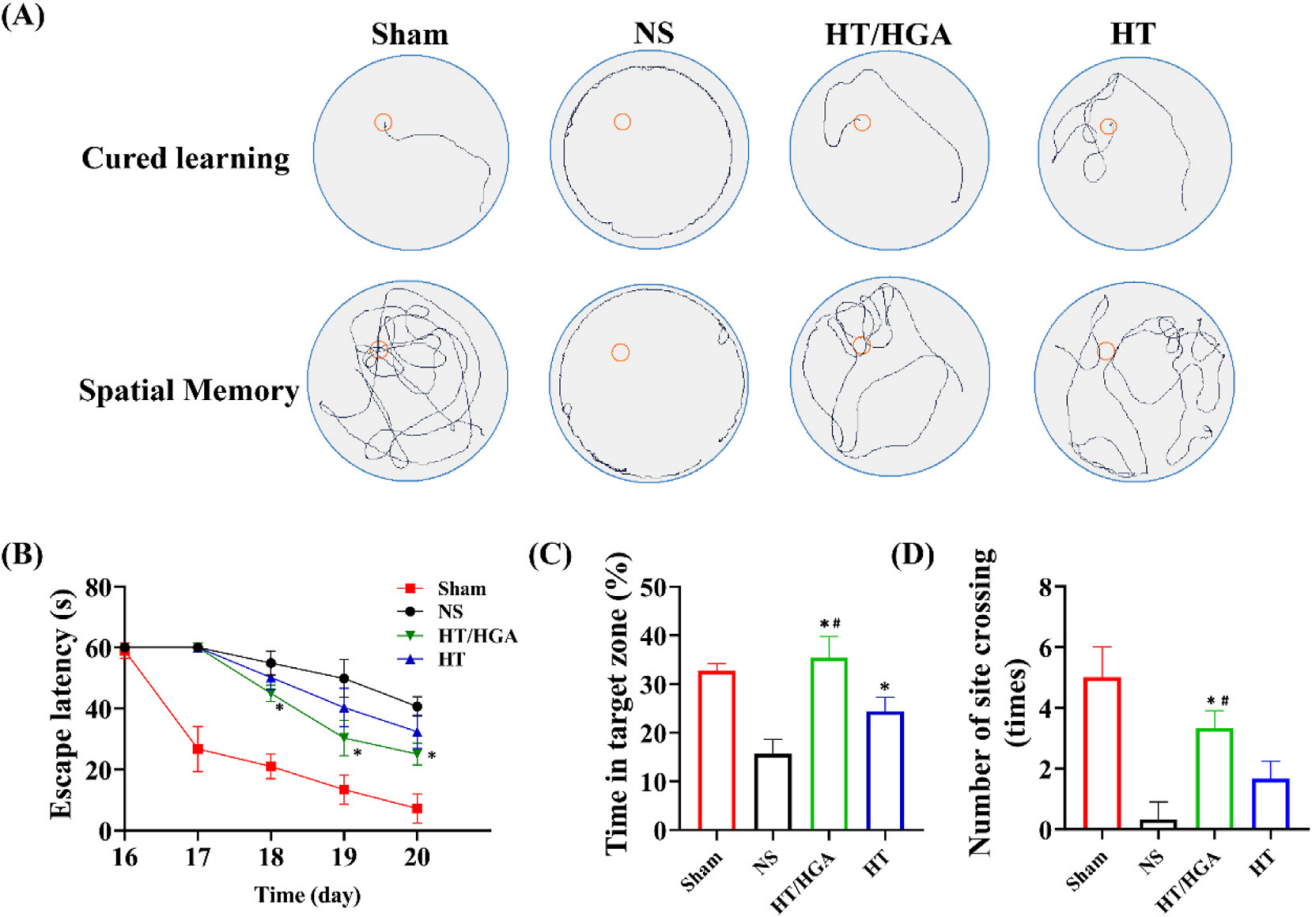
The learning and memory ability evaluated by MWM behavior test. (A) Swimming trails; (B) escape latency; (C) time in target zone (I quadrant); (D) the number of platform crossings. (∗*p*＜0.05, compared with NS group; ^#^*p*＜0.05, compared with HT hydrogel group, Mean ​± ​SD, n ​= ​6).

Following TBI, the production of ROS and a variety of proinflammatory cytokines storm will be triggered, subsequent cascade of signaling causes neuronal damage and ultimately disrupts the structure and brain function, which brings different neurological and pathological conditions. Morris water maze (MWM) is the gold standard behavioral test to assess cognitive deficits in rodents [66]. MWM test was performed on day 16–21 post-implantation in our study as shown in Fig. 9A. For the spatial navigation trial, HT group displayed a shorter escape time and the escape latency in HT/HGA group was notably reduced compared with NS group (Fig. 9B), indicating that hydrogels implantation could improve the learning ability of TBI mice. For positioning navigational trial, a marked increase of time in the target zone (I quadrant) was observed in HT and HT/HGA groups compared with NS group, and there was an obvious difference between HT and HT/HGA groups (Fig. 9C). In addition, as shown in Fig. 9D, HT/HGA group exhibited a clearly increased crossing number than NS and HT groups (*p* ​< ​0.05). These results indicated that HT/HGA hydrogel treatment improved the learning and memory ability after TBI.

### 3.10 HT/HGA hydrogel enhanced the neurogenesis and brain tissue remodeling after TBI

**Fig. 10:**
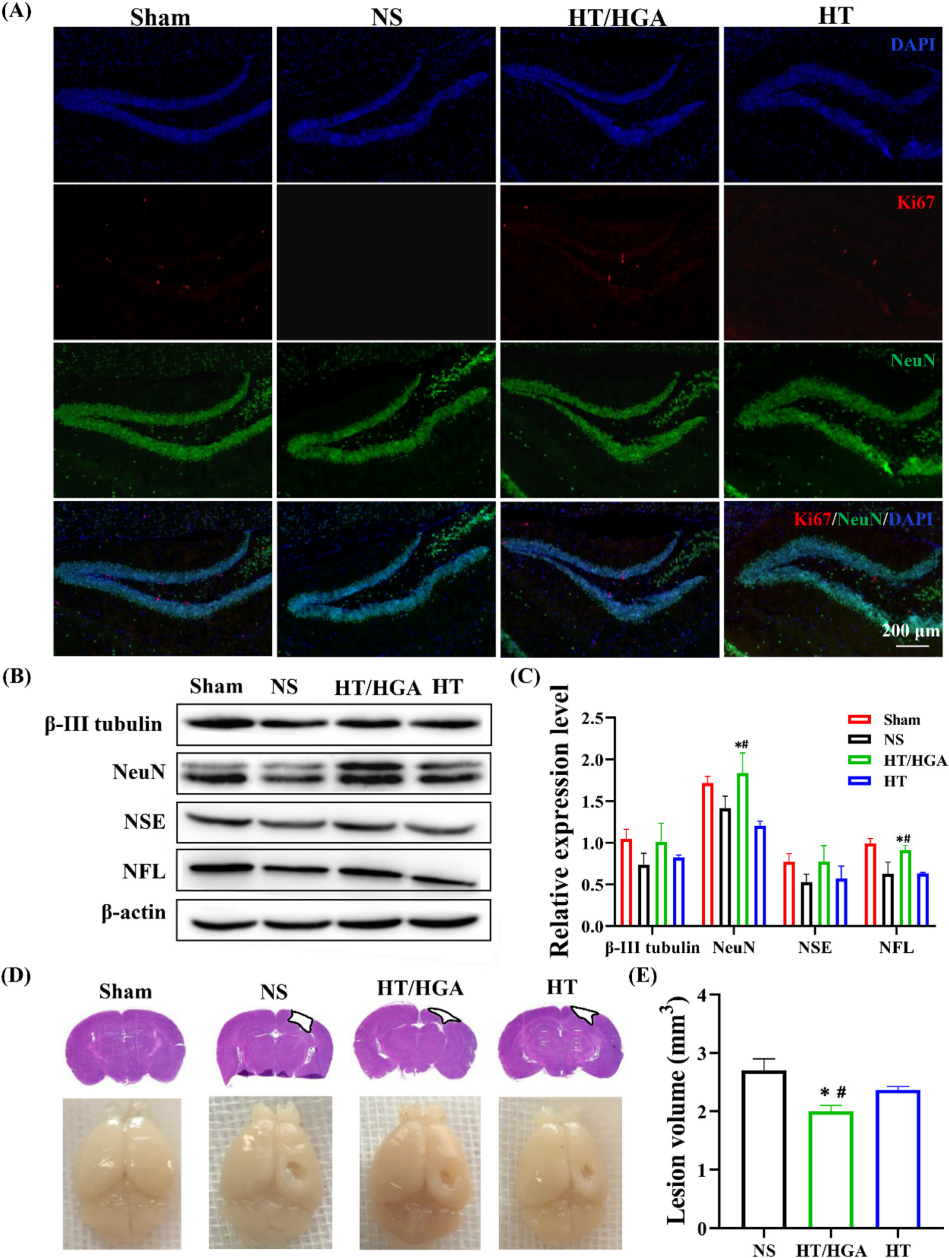
Neurogenesis and brain tissue remodeling after treatment for 21 days. (A) Immunofluorescence staining of NeuN and Ki67; (B) western blot and (C) relative expression of β-III tubulin, NeuN, NSE and NFL; (D) H&E staining and imaging of TBI mice brain; (E) brain lesion volume. (∗*p*＜0.05, compared with NS group; ^#^*p*＜0.05, compared with HT hydrogel group, Mean ​± ​SD, n ​= ​3).

Abnormal hippocampal activity such as dysregulated cellular homeostasis, dysfunctional synaptic neurotransmission, regional changes in circuit excitability, a decrease in intrinsic hippocampal theta oscillations, disruption of the theta rhythm and synaptic function, finally resulting neuronal cell death and loss [67], thus can lead to learning and memory dysfunction. Therefore, we speculated that neurogenesis may involve with the process of neural function recovery by HT/HGA hydrogel treatment. To verify our hypothesis, immunofluorescence was performed to examine the neurogenesis in hippocampus (Fig. 10A). NS group displayed less Ki67 (proliferation-specific marker) and NeuN (neuron-specific marker) positive cells in DG zone, indicating that the neurogenesis was significantly affected by TBI. HT and HT/HGA hydrogels treatment evidently increased Ki67 and NeuN-positive cells after implantation for 21 days. We further examined the expression of neuron-specific proteins by western blot. As shown in Fig. 10B and C, compared with NS and HT groups, HT/HGA hydrogel treatment up-regulated the expression of β-III tubulin, NeuN, NSE and NFL at day 21, and the expression of NeuN and NFL both had significance (*p* ​< ​0.05). Moreover, H&E staining and digital camera imaging (Fig. 10D) were carried out to investigate the brain tissue remodeling. As shown in Fig. 10E, compared with NS group, damaged area in HT and HT/HGA groups was smaller, and HT/HGA group was the lowest, indicating better brain structure recovery and reconstruction following treatment. These results suggested that HT/HGA hydrogel implantation significantly enhanced the neural cell survival and neurogenesis.

### 3.11 *In vivo* toxicity analysis of HT/HGA hydrogel

**Fig. 11:**
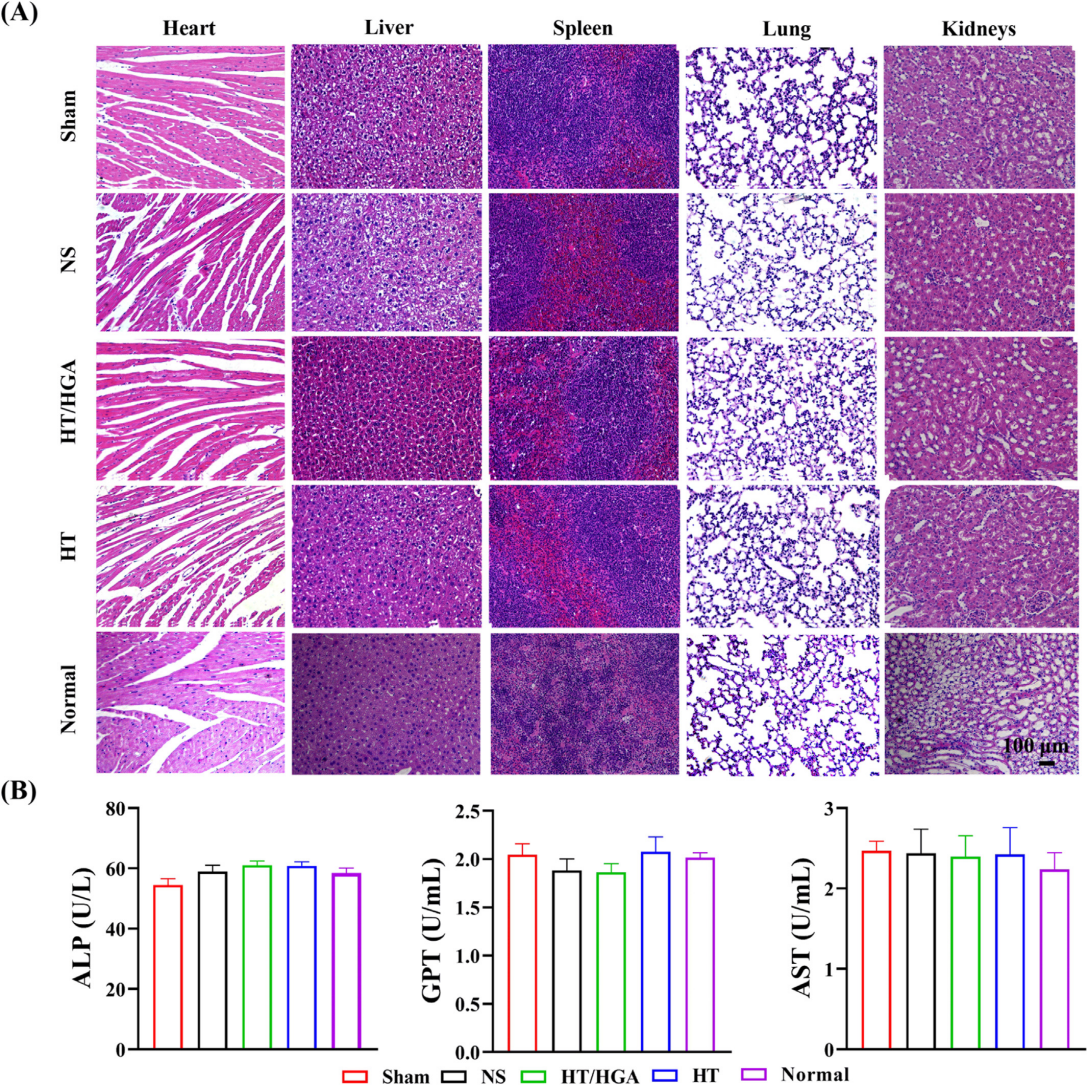
Systemic toxicity analysis. (A) H&E staining of heart, liver, spleen, lung and kidney; (B) blood biochemistry tests of ALP, GPT and AST activities in all groups after treatment for 21 days.

Finally, we further examined the systemic biosafety of HT/HGA hydrogel on vital organs including heart, liver, spleen, lung, and kidney by H&E staining. According to the results in Fig. 11A, H&E staining of Sham, NS, HT and HT/HGA groups showed that there were no noticeable changes compared with that of Normal group, indicating that this biomaterial had no obvious biological toxicity. In addition, there was no significant difference in ALP, GPT and AST activities among all groups (Fig. 11B), indicating that hydrogel had no obvious hepatotoxicity.

## 4 Conclusion

In this study, we developed a novel functional injectable hyaluronic acid hydrogel HT/HGA with free radicals scavenging activity. By dual-enzymatically crosslinking reaction, this hydrogel was formed *in situ* and highly biocompatible both *in vitro* and *in vivo*. Our study showed that the incorporation of HGA conjugate highly increased the hydroxyl and DPPH radicals scavenging efficiency compared to HT hydrogel alone. In addition, the HT/HGA hydrogel could suppress intracellular ROS production and protect N2a and HT22 ​cells from the damage of H_2_O_2_-induced oxidative stress. Particularly, HT/HGA hydrogel implantation reduced the oxidative stress through activation of Nrf2/HO-1 signaling pathway, suppressed the expression of pro-inflammatory factors and promoted the polarization of microglia from M1 to M2 phenotypes, finally protecting BBB integrity, enhancing neurogenesis and improving neural function recovery including motor, learning and memory after TBI. Given all these features, the HT/HGA hydrogel developed in our study provides a feasible therapeutic strategy aiming to TBI in mitigating the secondary injury mainly caused by oxidative stress and neuroinflammation.

## Credit author statement

**Dan Zhang**: Resources, Writing – original draft, preparation. **Yikun Ren**: Writing – review & editing. **Yuanmeng He**: Resources. **Rong Chang**: Methodology. **Shen Guo**: Writing – review & editing. **Shanshan Ma:** Writing – review & editing. **Fangxia Guan**: Conceptualization, Writing – review & editing. **Minghao Yao**: Conceptualization, Validation, Investigation, Writing – review & editing.

## Declaration of competing interest

The authors declare that they have no known competing financial interests or personal relationships that could have appeared to influence the work reported in this paper.

## References

[1] Buchmann Godinho D., da Silva Fiorin F., Schneider Oliveira M., Furian A.F., Rechia Fighera M., Freire Royes L.F. (2021). The immunological influence of physical exercise on TBI-induced pathophysiology: crosstalk between the spleen, gut, and brain. Neurosci. Biobehav. Rev..

[2] Peruzzaro S.T., Andrews M.M.M., Al-Gharaibeh A., Pupiec O., Resk M., Story D., Maiti P., Rossignol J., Dunbar G.L. (2019). Transplantation of mesenchymal stem cells genetically engineered to overexpress interleukin-10 promotes alternative inflammatory response in rat model of traumatic brain injury. J. Neuroinflammation.

[3] Hanscom M., Loane D.J., Shea-Donohue T. (2021). Brain-gut axis dysfunction in the pathogenesis of traumatic brain injury. J. Clin. Invest..

[4] Liu Y.W., Li S., Dai S.S. (2018). Neutrophils in traumatic brain injury (TBI): friend or foe?. J. Neuroinflammation.

[5] Kandell R.M., Kudryashev J.A., Kwon E.J. (2021). Targeting the extracellular matrix in traumatic brain injury increases signal generation from an activity-based nanosensor. ACS Nano.

[6] Kayambankadzanja R.K., Samwel R., Baker T. (2022). Pragmatic sedation strategies to prevent secondary brain injury in low-resource settings. Anaesthesia.

[7] Hood E.D., Chorny M., Greineder C.F., I S.A., Levy R.J., Muzykantov V.R. (2014). Endothelial targeting of nanocarriers loaded with antioxidant enzymes for protection against vascular oxidative stress and inflammation. Biomaterials.

[8] Wang X., Rivera-Bolanos N., Jiang B., Ameer G.A. (2019). Advanced functional biomaterials for stem cell delivery in regenerative engineering and medicine. Adv. Funct. Mater..

[9] Zheng Y., Wu G., Chen L., Zhang Y., Luo Y., Zheng Y., Hu F., Forouzanfar T., Lin H., Liu B. (2021). Neuro-regenerative imidazole-functionalized GelMA hydrogel loaded with hAMSC and SDF-1alpha promote stem cell differentiation and repair focal brain injury. Bioact. Mater..

[10] Moeinabadi-Bidgoli K., Babajani A., Yazdanpanah G., Farhadihosseinabadi B., Jamshidi E., Bahrami S., Niknejad H. (2021). Translational insights into stem cell preconditioning: from molecular mechanisms to preclinical applications. Biomed. Pharmacother..

[11] Lutton E.M., Razmpour R., Andrews A.M., Cannella L.A., Son Y.J., Shuvaev V.V., Muzykantov V.R., Ramirez S.H. (2017). Acute administration of catalase targeted to ICAM-1 attenuates neuropathology in experimental traumatic brain injury. Sci. Rep..

[12] Kartha S., Yan L., Weisshaar C.L., Ita M.E., Shuvaev V.V., Muzykantov V.R., Tsourkas A., Winkelstein B.A., Cheng Z. (2017). Superoxide dismutase-loaded porous polymersomes as highly efficient antioxidants for treating neuropathic pain. Adv. Healthc. Mater..

[13] Zhu Y., Wang H., Fang J., Dai W., Zhou J., Wang X., Zhou M. (2018). SS-31 provides neuroprotection by reversing mitochondrial dysfunction after traumatic brain injury. Oxid. Med. Cell. Longev..

[14] Roth T.L., Nayak D., Atanasijevic T., Koretsky A.P., Latour L.L., McGavern D.B. (2014). Transcranial amelioration of inflammation and cell death after brain injury. Nature.

[15] Sharma R., Kambhampati S.P., Zhang Z., Sharma A., Chen S., Duh E.I., Kannan S., Tso M.O.M., Kannan R.M. (2020). Dendrimer mediated targeted delivery of sinomenine for the treatment of acute neuroinflammation in traumatic brain injury. J. Contr. Release.

[16] Lagraoui M., Sukumar G., Latoche J.R., Maynard S.K., Dalgard C.L., Schaefer B.C. (2017). Salsalate treatment following traumatic brain injury reduces inflammation and promotes a neuroprotective and neurogenic transcriptional response with concomitant functional recovery. Brain Behav. Immun..

[17] Mu X., He H., Wang J., Long W., Li Q., Liu H., Gao Y., Ouyang L., Ren Q., Sun S., Wang J., Yang J., Liu Q., Sun Y., Liu C., Zhang X.D., Hu W. (2019). Carbogenic nanozyme with ultrahigh reactive nitrogen species selectivity for traumatic brain injury. Nano Lett..

[18] Mu X., Wang J., He H., Li Q., Yang B., Wang J., Liu H., Gao Y., Ouyang L., Sun S., Ren Q., Shi X., Hao W., Fei Q., Yang J., Li L., Vest R., Wyss-Coray T., Luo J., Zhang X.D. (2021). An oligomeric semiconducting nanozyme with ultrafast electron transfers alleviates acute brain injury. Sci. Adv..

[19] He H., Shi X., Wang J., Wang X., Wang Q., Yu D., Ge B., Zhang X., Huang F. (2020). Reactive oxygen species-induced aggregation of nanozymes for neuron injury. ACS Appl. Mater. Interfaces.

[20] Liu H., Li Y., Sun S., Xin Q., Liu S., Mu X., Yuan X., Chen K., Wang H., Varga K., Mi W., Yang J., Zhang X.D. (2021). Catalytically potent and selective clusterzymes for modulation of neuroinflammation through single-atom substitutions. Nat. Commun..

[21] Guan P., Liu C., Xie D., Mao S., Ji Y., Lin Y., Chen Z., Wang Q., Fan L., Sun Y. (2022). Exosome-loaded extracellular matrix-mimic hydrogel with anti-inflammatory property Facilitates/promotes growth plate injury repair. Bioact. Mater..

[22] Qian F., Han Y., Han Z., Zhang D., Zhang L., Zhao G., Li S., Jin G., Yu R., Liu H. (2021). In Situ implantable, post-trauma microenvironment-responsive, ROS Depletion Hydrogels for the treatment of Traumatic brain injury. Biomaterials.

[23] Kuan C.-Y., Lin Y.-Y., Chen C.-Y., Yang C.-C., Chi C.-Y., Li C.-H., Dong G.-C., Lin F.-H. (2019). The preparation of oxidized methylcellulose crosslinked by adipic acid dihydrazide loaded with vitamin C for traumatic brain injury. J. Mater. Chem. B.

[24] Ashrafizadeh M., Zarrabi A., Mirzaei S., Hashemi F., Samarghandian S., Zabolian A., Hushmandi K., Ang H.L., Sethi G., Kumar A.P., Ahn K.S., Nabavi N., Khan H., Makvandi P., Varma R.S. (2021). Gallic acid for cancer therapy: molecular mechanisms and boosting efficacy by nanoscopical delivery. Food Chem. Toxicol..

[25] Wang H., You S., Wang W., Zeng Y., Su R., Qi W., Wang K., He Z. (2022). Laccase-catalyzed soy protein and gallic acid complexation: effects on conformational structures and antioxidant activity. Food Chem..

[26] Zhang X., Zhou D., Cao Y., Zhang Y., Xiao X., Liu F., Yu Y. (2022). Synergistic inactivation of Escherichia coli O157:H7 and Staphylococcus aureus by gallic acid and thymol and its potential application on fresh-cut tomatoes. Food Microbiol..

[27] Zhang Y., Xi K., Fu X., Sun H., Wang H., Yu D., Li Z., Ma Y., Liu X., Huang B., Wang J., Li G., Cui J., Li X., Ni S. (2021). Versatile metal-phenolic network nanoparticles for multitargeted combination therapy and magnetic resonance tracing in glioblastoma. Biomaterials.

[28] Lin Y., Luo T., Weng A., Huang X., Yao Y., Fu Z., Li Y., Liu A., Li X., Chen D., Pan H. (2020). Gallic acid alleviates gouty arthritis by inhibiting NLRP3 inflammasome activation and pyroptosis through enhancing Nrf2 signaling. Front. Immunol..

[29] Bai J., Zhang Y., Tang C., Hou Y., Ai X., Chen X., Zhang Y., Wang X., Meng X. (2021). Gallic acid: pharmacological activities and molecular mechanisms involved in inflammation-related diseases. Biomed. Pharmacother..

[30] Park S.G., Li M.X., Cho W.K., Joung Y.K., Huh K.M. (2021). Thermosensitive gallic acid-conjugated hexanoyl glycol chitosan as a novel wound healing biomaterial. Carbohydr. Polym..

[31] Thi P.L., Lee Y., Tran D.L., Thi T.T.H., Kang J.I., Park K.M., Park K.D. (2020). In situ forming and reactive oxygen species-scavenging gelatin hydrogels for enhancing wound healing efficacy. Acta Biomater..

[32] Wang L., Li J., Zhang D., Ma S., Zhang J., Gao F., Guan F., Yao M. (2020). Dual-enzymatically crosslinked and injectable hyaluronic acid hydrogels for potential application in tissue engineering. RSC Adv..

[33] Basha S.I., Ghosh S., Vinothkumar K., Ramesh B., Kumari P.H.P., Mohan K.V.M., Sukumar E. (2020). Fumaric acid incorporated Ag/agar-agar hybrid hydrogel: a multifunctional avenue to tackle wound healing. Mater. Sci. Eng. Mater. Biol. Appl..

[34] Grenier A., Legault J., Pichette A., Jean L., Belanger A., Pouliot R. (2021). Antioxidant, anti-inflammatory, and anti-aging potential of a kalmia angustifolia extract and identification of some major compounds. Antioxidants.

[35] Yao M., Li J., Zhang J., Ma S., Wang L., Gao F., Guan F. (2021). Dual-enzymatically cross-linked gelatin hydrogel enhances neural differentiation of human umbilical cord mesenchymal stem cells and functional recovery in experimental murine spinal cord injury. J. Mater. Chem. B.

[36] Xie B.S., Wang Y.Q., Lin Y., Mao Q., Feng J.F., Gao G.Y., Jiang J.Y. (2019). Inhibition of ferroptosis attenuates tissue damage and improves long-term outcomes after traumatic brain injury in mice. CNS Neurosci. Ther..

[37] Wang D., Xu X., Wu Y.G., Lyu L., Zhou Z.W., Zhang J.N. (2018). Dexmedetomidine attenuates traumatic brain injury: action pathway and mechanisms. Neural Regen Res..

[38] Yao M., Gao F., Xu R., Zhang J., Chen Y., Guan F. (2019). A dual-enzymatically cross-linked injectable gelatin hydrogel loaded with BMSC improves neurological function recovery of traumatic brain injury in rats. Biomater. Sci..

[39] Wu H., Wang R., Qin X., Liu D., Wang W., Xu J., Jiang H., Pan F. (2021). Effects of chronic stress on depressive-like behaviors and JMJD3 expression in the prefrontal cortex and hippocampus of C57BL/6 and ob/ob mice. J. Psychiatr. Res..

[40] Li J., Zhang D., Guo S., Zhao C., Wang L., Ma S., Guan F., Yao M. (2021). Dual-enzymatically cross-linked gelatin hydrogel promotes neural differentiation and neurotrophin secretion of bone marrow-derived mesenchymal stem cells for treatment of moderate traumatic brain injury. Int. J. Biol. Macromol..

[41] Park H.H., Ko S.C., Oh G.W., Jang Y.M., Kim Y.M., Park W.S., Choi I.W., Jung W.K. (2018). Characterization and biological activity of PVA hydrogel containing chitooligosaccharides conjugated with gallic acid. Carbohydr. Polym..

[42] Gao F., Li J., Wang L., Zhang D., Zhang J., Guan F., Yao M.H. (2020). Dual-enzymatically crosslinked hyaluronic acid hydrogel as a long-time 3D stem cell culture system. Biomed. Mater..

[43] Tran D.L., Le Thi P., Hoang Thi T.T., Park K.D. (2020). Novel enzymatically crosslinked chitosan hydrogels with free-radical-scavenging property and promoted cellular behaviors under hyperglycemia. Prog. Nat. Sci.: Mater. Int..

[44] Godbe J.M., Freeman R., Burbulla L.F., Lewis J., Krainc D., Stupp S.I. (2020). Gelator length precisely tunes supramolecular hydrogel stiffness and neuronal phenotype in 3D culture. ACS Biomater. Sci. Eng..

[45] Pisoschi A.M., Pop A., Iordache F., Stanca L., Predoi G., Serban A.I. (2021). Oxidative stress mitigation by antioxidants - an overview on their chemistry and influences on health status. Eur. J. Med. Chem..

[46] Chandrasekhar Y., Phani Kumar G., Ramya E.M., Anilakumar K.R. (2018). Gallic acid protects 6-OHDA induced neurotoxicity by attenuating oxidative stress in human dopaminergic cell line. Neurochem. Res..

[47] Carvajal F.J., Cerpa W. (2021). Regulation of phosphorylated state of NMDA receptor by STEP61 phosphatase after mild-traumatic brain injury: role of oxidative stress. Antioxidants.

[48] Lohan S.B., Ivanov D., Schuler N., Berger B., Zastrow L., Lademann J., Meinke M.C. (2021). Switching from healthy to unhealthy oxidative stress - does the radical type can be used as an indicator?. Free Radic. Biol. Med..

[49] Angelova P.R., Esteras N., Abramov A.Y. (2021). Mitochondria and lipid peroxidation in the mechanism of neurodegeneration: finding ways for prevention. Med. Res. Rev..

[50] Lin C.H., Lane H.Y. (2021). Plasma glutathione levels decreased with cognitive decline among people with mild cognitive impairment (MCI): a two-year prospective study. Antioxidants.

[51] Higashi Y., Aratake T., Shimizu T., Shimizu S., Saito M. (2021). Protective role of glutathione in the Hippocampus after brain ischemia. Int. J. Mol. Sci..

[52] Feng R.B., Wang Y., He C., Yang Y., Wan J.B. (2018). Gallic acid, a natural polyphenol, protects against tert-butyl hydroperoxide- induced hepatotoxicity by activating ERK-Nrf2-Keap1-mediated antioxidative response. Food Chem. Toxicol..

[53] Zhang Z., Peng L., Fu Y., Wang W., Wang P., Zhou F. (2021). Ginnalin A binds to the subpockets of Keap1 kelch domain to activate the nrf2-regulated antioxidant defense system in SH-SY5Y cells. ACS Chem. Neurosci..

[54] Ronaldson P.T., Davis T.P. (2020). Regulation of blood-brain barrier integrity by microglia in health and disease: a therapeutic opportunity. J. Cerebr. Blood Flow Metabol..

[55] Chen H., Guan B., Chen S., Yang D., Shen J. (2021). Peroxynitrite activates NLRP3 inflammasome and contributes to hemorrhagic transformation and poor outcome in ischemic stroke with hyperglycemia. Free Radic. Biol. Med..

[56] Lee T.H., Chen J.L., Liu P.S., Tsai M.M., Wang S.J., Hsieh H.L. (2020). Rottlerin, a natural polyphenol compound, inhibits upregulation of matrix metalloproteinase-9 and brain astrocytic migration by reducing PKC-delta-dependent ROS signal. J. Neuroinflammation.

[57] Liu Y., Wei H., Tang J., Yuan J., Wu M., Yao C., Hosoi K., Yu S., Zhao X., Han Y., Chen G. (2020). Dysfunction of pulmonary epithelial tight junction induced by silicon dioxide nanoparticles via the ROS/ERK pathway and protein degradation. Chemosphere.

[58] Yamamura H., Suzuki Y., Asai K., Imaizumi Y., Yamamura H. (2020). Oxidative stress facilitates cell death by inhibiting Orai1-mediated Ca(2+) entry in brain capillary endothelial cells. Biochem. Biophys. Res. Commun..

[59] Lutton E.M., Farney S.K., Andrews A.M., Shuvaev V.V., Chuang G.Y., Muzykantov V.R., Ramirez S.H. (2019). Endothelial targeted strategies to combat oxidative stress: improving outcomes in traumatic brain injury. Front. Neurol..

[60] Hussain T., Tan B., Yin Y., Blachier F., Tossou M.C., Rahu N. (2016). Oxidative stress and inflammation: what polyphenols can do for us?. Oxid. Med. Cell. Longev..

[61] Shi K., Zhang J., Dong J.F., Shi F.D. (2019). Dissemination of brain inflammation in traumatic brain injury. Cell. Mol. Immunol..

[62] Wu Y., Jiang H., Chen G., Chen X., Hu C., Su X., Tan F., Zhao X. (2021). Preventive effect of gonggan (citrus reticulata blanco var. Gonggan) peel extract on ethanol/HCl-induced gastric injury in mice via an anti-oxidative mechanism. Front. Pharmacol..

[63] Xiao H.X., Song B., Li Q., Shao Y.M., Zhang Y.B., Chang X.L., Zhou Z.J. (2022). Paraquat mediates BV-2 microglia activation by raising intracellular ROS and inhibiting Akt1 phosphorylation. Toxicol. Lett..

[64] Shen H., Xu B., Yang C., Xue W., You Z., Wu X., Ma D., Shao D., Leong K., Dai J. (2022). A DAMP-scavenging, IL-10-releasing hydrogel promotes neural regeneration and motor function recovery after spinal cord injury. Biomaterials.

[65] Deng W., Fan C., Shen R., Wu Y., Du R., Teng J. (2020). Long noncoding MIAT acting as a ceRNA to sponge microRNA-204-5p to participate in cerebral microvascular endothelial cell injury after cerebral ischemia through regulating HMGB1. J. Cell. Physiol..

[66] Tucker L.B., Velosky A.G., McCabe J.T. (2018). Applications of the Morris water maze in translational traumatic brain injury research. Neurosci. Biobehav. Rev..

[67] Liang J., Wu Y., Yuan H., Yang Y., Xiong Q., Liang C., Li Z., Li C., Zhang G., Lai X., Hu Y., Hou S. (2019). Dendrobium officinale polysaccharides attenuate learning and memory disabilities via anti-oxidant and anti-inflammatory actions. Int. J. Biol. Macromol..

