# Supplementary material for: In situ forming and biocompatible hyaluronic acid hydrogel with reactive oxygen species-scavenging activity to improve traumatic brain injury repair by suppressing oxidative stress and neuroinflammation
Source: Mater Today Bio. 2022 May 10;15:100278. doi: 10.1016/j.mtbio.2022.100278 (PMC9119840; doi:10.1016/j.mtbio.2022.100278)
Supplement: Supplementary file 1 [file mmc1.docx]

In situ forming **and biocompatible hyaluronic acid hydrogel with reactive oxygen species-scavenging activity to improve traumatic brain injury repair by suppressing oxidative stress and neuroinflammation**

*Dan Zhang, Yikun Ren, Yuanmeng He, Rong Chang, Shen Guo, Shanshan Ma, Fangxia Guan^*^, Minghao Yao^*^*

School of Life Science, Zhengzhou University, 100 Science Road, Zhengzhou 450001, P. R. China

^*^ E-mail: yao453343550@126.com, guanfangxia@126.com


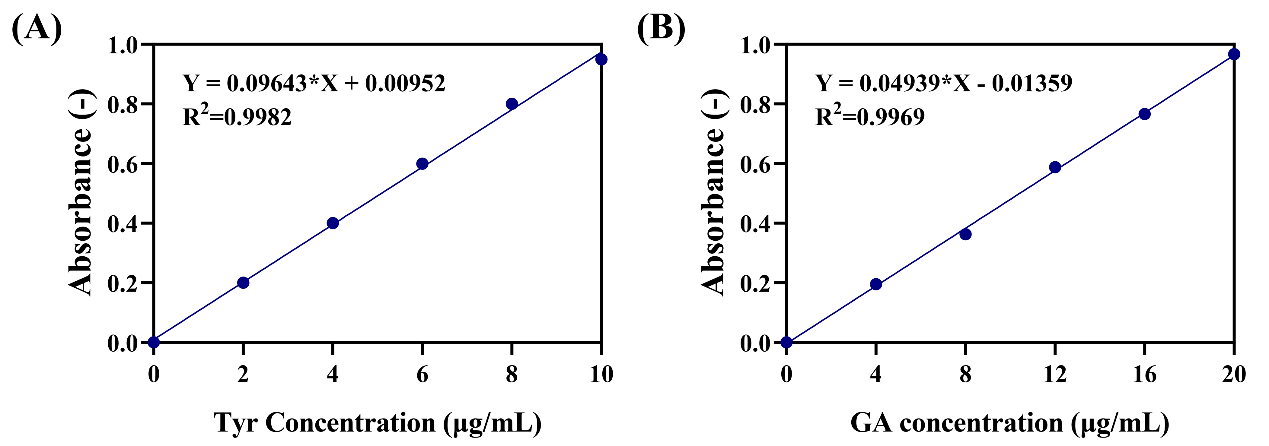


Figure S1 Standard curves of Tyr (A) and GA (B) for UV-vis spectra. (Mean ± SD, n=3)


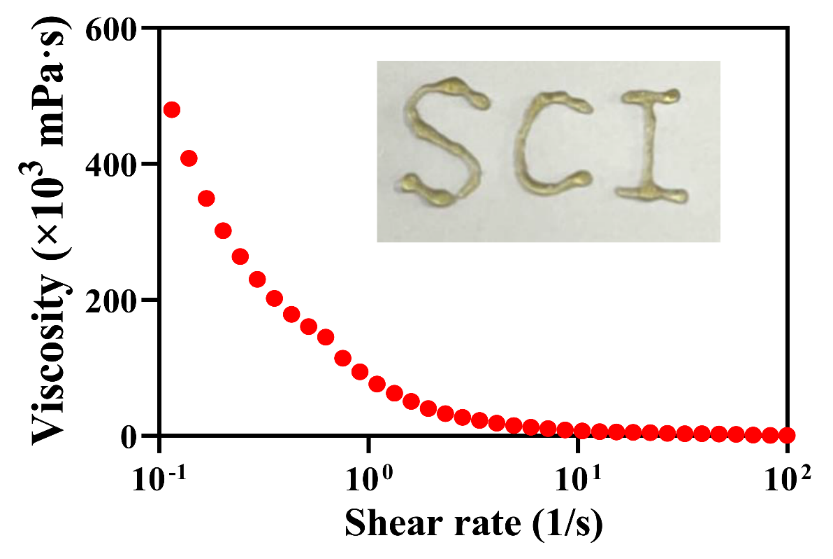


Figure S2 Shear-thinning property of HT_0.5_HGA_0.5_ hydrogel. Insert: Presentation of injectability of the hydrogel.


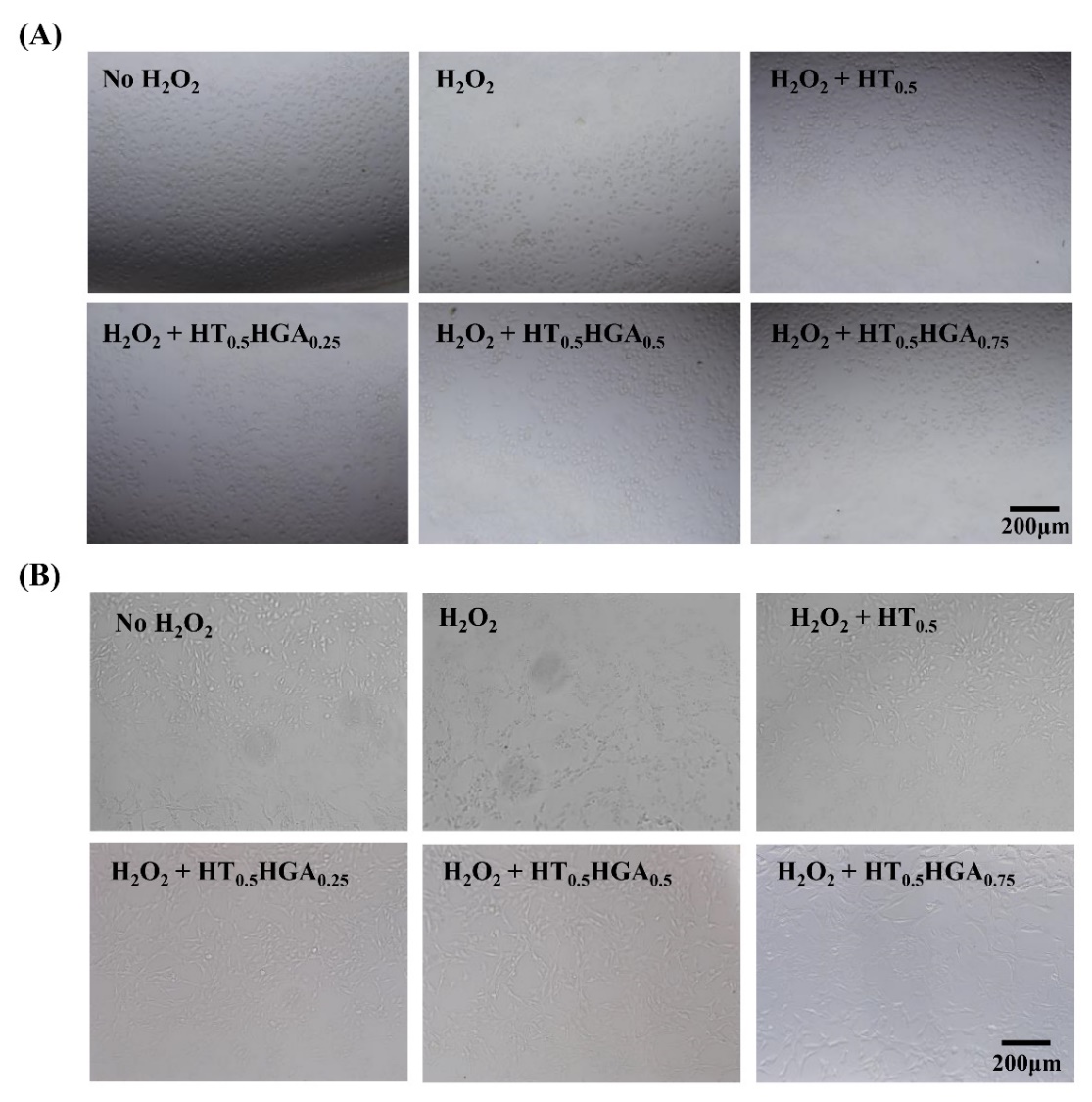


Figure S3 Cellular morphology of N2a (A) and HT22 cells (B) with or without hydrogels in H_2_O_2_-induced microenvironment.


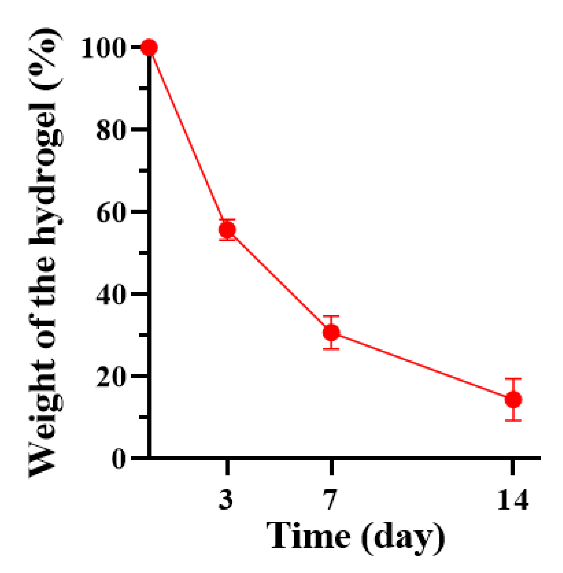


Figure S4 Quantitative analysis of weight of HT_0.5_HGA_0.5_ hydrogel when it was injected subcutaneously. (Mean ± SD, n=3)


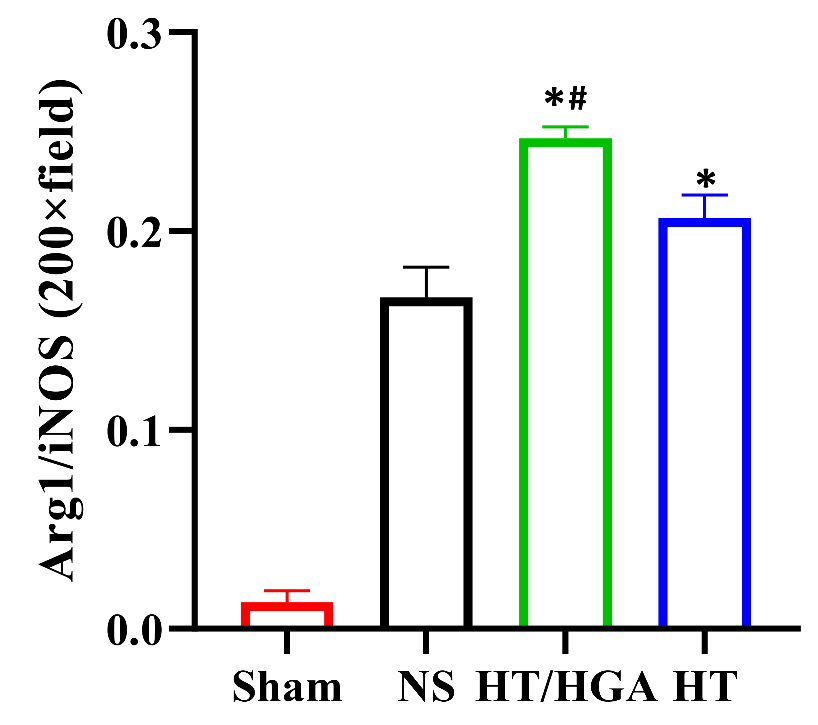


Figure S5 iNOS/Arg1 ratio statistics from the results of immunofluorescence staining. (**p*＜0.05, compared with NS group; ^#^*p*＜0.05, compared with HT hydrogel group, Mean ± SD, n=3)
